# Trajectory and uniqueness of mutational signatures in yeast mutators

**DOI:** 10.1073/pnas.2011332117

**Published:** 2020-09-23

**Authors:** Sophie Loeillet, Mareike Herzog, Fabio Puddu, Patricia Legoix, Sylvain Baulande, Stephen P. Jackson, Alain G. Nicolas

**Affiliations:** ^a^Institut Curie, Paris Sciences et Lettres Research University, CNRS, UMR3244, 75248 Paris Cedex 05, France;; ^b^Sorbonne Universités, Université Pierre et Marie Curie Paris 06, CNRS, UMR3244, 75248 Paris Cedex 05, France;; ^c^Wellcome/Cancer Research UK Gurdon Institute and Department of Biochemistry, Cambridge CB2 1QN, United Kingdom;; ^d^ICGex NGS Platform, Institut Curie, 75248 Paris Cedex 05, France

**Keywords:** mutator genes, mutational profiles, Pol zeta, loss of heterozygosity, dynamics of mutation accumulation

## Abstract

The acquisition of mutations plays critical roles in adaptation, evolution, senescence, and tumorigenesis. Massive genome sequencing has allowed extraction of specific features of many mutational landscapes but it remains difficult to retrospectively determine the mechanistic origin(s), selective forces, and trajectories of transient or persistent mutations and genome rearrangements. Here, we conducted a prospective reciprocal approach to inactivate 13 single or multiple evolutionary conserved genes involved in distinct genome maintenance processes and characterize de novo mutations in 274 diploid *Saccharomyces cerevisiae* mutation accumulation lines. This approach revealed the diversity, complexity, and ultimate uniqueness of mutational landscapes, differently composed of base substitutions, small insertions/deletions (InDels), structural variants, and/or ploidy variations. Several landscapes parallel the repertoire of mutational signatures in human cancers while others are either novel or composites of subsignatures resulting from distinct DNA damage lesions. Notably, the increase of base substitutions in the homologous recombination-deficient Rad51 mutant, specifically dependent on the Polζ translesion polymerase, yields COSMIC signature 3 observed in BRCA1/BRCA2-mutant breast cancer tumors. Furthermore, “mutome” analyses in highly polymorphic diploids and single-cell bottleneck lineages revealed a diverse spectrum of loss-of-heterozygosity (LOH) signatures characterized by interstitial and terminal chromosomal events resulting from interhomolog mitotic cross-overs. Following the appearance of heterozygous mutations, the strong stimulation of LOHs in the *rad27/FEN1* and *tsa1/PRDX1* backgrounds leads to fixation of homozygous mutations or their loss along the lineage. Overall, these mutomes and their trajectories provide a mechanistic framework to understand the origin and dynamics of genome variations that accumulate during clonal evolution.

## Significance

Deficiencies in genome maintenance genes result in increased mutagenesis and genome rearrangements that impact cell viability, species adaptation, and evolvability. The accumulation of somatic mutations is also a landmark of most tumor cells but it remains difficult to retrospectively determine their mechanistic origin(s). Here, we conducted a prospective reciprocal approach to inactivate evolutionary conserved genes involved in various genome maintenance processes and characterize de novo mutations in diploid *S. cerevisiae* mutation accumulation lines. Our results revealed the diversity, trajectory, complexity, and ultimate uniqueness of the clonal mutational landscapes. Some mutational signatures resemble those found in human tumors.

Acquired and transitory mutations, broadly genome instability, can be evolutionarily advantageous in contributing to the adaptation of species in changing environments, or detrimental in reducing short- and long-term fitness (1–3). Mechanistically, spontaneous mutations in normal cells, exposure to environmental genotoxic compounds, and deficiencies in genome maintenance genes are prominent sources of subtle or drastic genome changes/rearrangements and eventually functional and phenotypic variations (4, 5). A paradigm for this phenomenon is the accumulation of a variable burden of passenger and driver somatic mutations in tumor cell lineages (6–11). Thus, genome sequencing and mutational landscape analyses of germline and somatic mutations have permitted the retrospective identification of the most likely environmental sources of mutagen exposures, such as ultraviolet light exposure in melanoma and smoking in lung cancers, or genetic features, such as deficiency in DNA mismatch repair in colon cancers and homologous recombination (HR) defects in breast and ovarian cancers (6–14). However, it remains puzzling that in numerous instances an environmental factor and/or defective mutator gene(s) are not found, although numerous relevant and evolutionarily conserved genome maintenance genes and pathways are known (5, 15, 16). Here, we conducted a reciprocal functional approach to inactivate one or several genes involved in distinct genome maintenance processes (replication, repair, recombination, oxidative stress response, or cell-cycle progression) in *Saccharomyces cerevisiae* diploids, establish the genome-wide mutational landscapes of mutation accumulation (MA) lines, explore the underlying mechanisms, and characterize the dynamics of mutation accumulation (and disappearance) along single-cell bottleneck passages.

## Results and Discussion

### Variety of Mutational Landscapes.

**Fig. 1.**
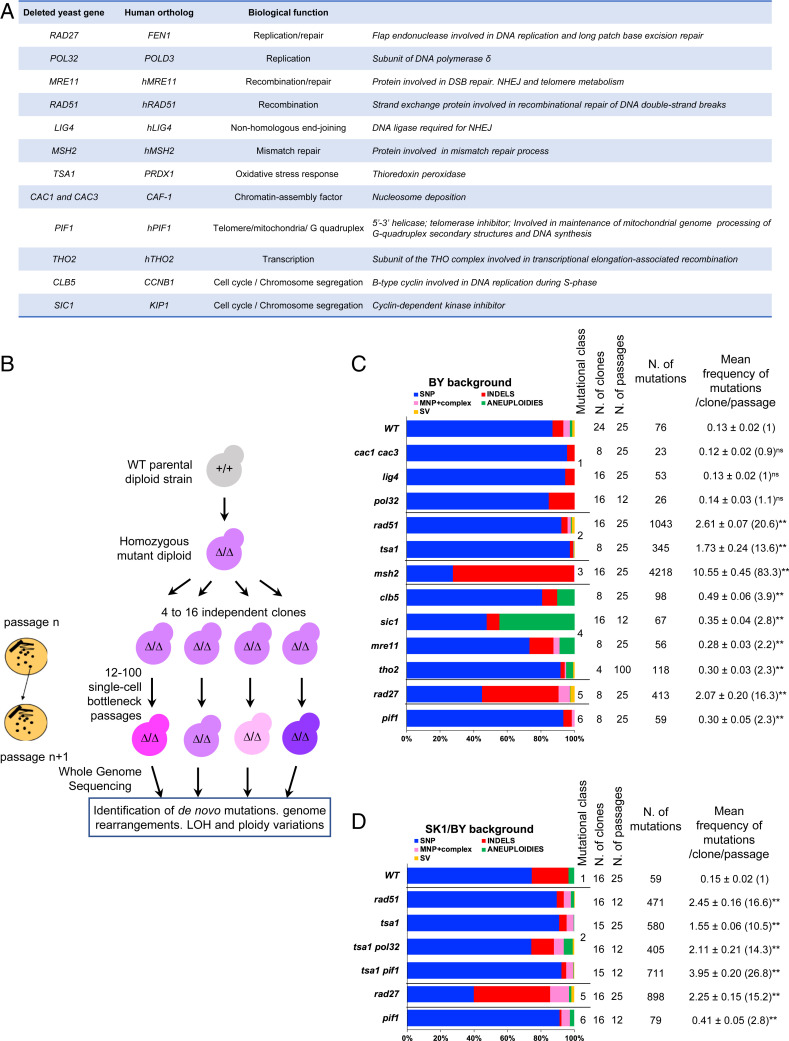
Mutational landscapes. (*A*) List of genes studied and their functions. (*B*) Experimental strategy to generate mutation accumulation lines. The WT diploid strains (BY/BY or SK1/BY background) were deleted for both copies (*∆/∆*) of the potential mutator gene(s). Then, 4 to 16 independent clones of the WT and *∆/∆* diploids were grown mitotically and derived for up to 100 single-cell bottleneck passages on YPD-rich medium at 30 °C (23). The genome of the resulting accumulation lines was individually sequenced by NGS and the reads were analyzed for detection of de novo mutations and genome rearrangements (*SI Appendix*, *Materials and Methods* and Fig. S1). (*C* and *D*) Mutational profiles in BY/BY and SK1/BY strains, respectively. N. of mutations: total number of de novo mutations detected in each strain, including single-nucleotide variants, small InDels, multinucleotide variants, “complex” events referring to combinations of SNPs and small InDels, chromosome aneuploidies, and structural variants (large deletions/insertions). The SNPs and small InDels comprise both heterozygous (allelic ratio ∼0.5) and apparently homozygous events (allelic ratio ∼1.0). For each mutant, the class of mutator profile and number of clones, passages, and mutations are indicated. The mean number of mutations per clone normalized to the number of passages and the SE are shown. The mutational fold variation compared with the corresponding WT is shown in parentheses. The Mann–Whitney–Wilcoxon test was performed to compare each mutant with WT (ns, not significant; **P < 0.01).

Overall, we established the mutational landscapes of 274 MA lines generated in the isogenic BY and/or hybrid SK1/BY wild-type (WT) backgrounds. Strains assessed included WT, 11 single-deletion mutants (hereafter abbreviated by gene name), and 3 double mutants covering various genome maintenance processes. Compared with WT, we analyzed the following mutant strains: *pif1*Δ, *pol32*Δ, and *rad27*Δ (replication), *msh2*Δ (mismatch repair), *mre11*Δ, *rad51*Δ, and *tho2*Δ (recombination and repair), *lig4*Δ (nonhomologous end joining), *tsa1*Δ (oxidative stress response), *cac1*Δ *cac3*Δ (nucleosome deposition), and *clb5*Δ and *sic1*Δ (cell-cycle progression) (https://www.yeastgenome.org/). Strain genotypes are indicated in Dataset S1. All of the genes assessed are evolutionarily conserved and most are implicated in human diseases and/or tumor development (Fig. 1*A*) (https://www.yeastgenome.org/, https://www.genecards.org/). To ensure the recovery of independent events, 4 to 16 individual colonies per strain were derived in parallel MA lines (Fig. 1*B*) and sequenced after a minimum of 180 single-cell bottleneck passages (see *SI Appendix*, *Materials and Methods* and Dataset S2 for individual clones). One passage corresponds to ∼25 generations. Our bioinformatics analyses of the next-generation sequencing (NGS) reads allowed identification of base substitutions (single-nucleotide polymorphisms; SNPs), multinucleotide polymorphisms (MNPs), small (1- to 44-bp) insertions/deletions (InDels), combinations of SNPs and small InDels (complexes), structural variants (SVs), as well as chromosomal ploidy variations and loss-of-heterozygosity (LOH) regions (*SI Appendix*, *Materials and Methods* and Fig. S1). The coordinates and annotations of the 8,876 de novo mutations identified in this study are reported in Datasets S3–S8. The number of mutations detected in the parallel MA lines of the same genotype was similar (*SI Appendix*, Fig. S2*A* and Dataset S9), thus excluding clonal effects. Except for a few common homopolymer InDels in *msh2* and *rad27* backgrounds, all mutations were different from one another.

Functionally, 4,103/5,416 (75.8%) base substitutions were located in a gene-coding region (Datasets S3 and S4), similar to random expectation (76.5%). At the protein level, 2,879 (53.2%) modified an amino acid with a presumptive moderate functional impact according to SnpEff annotation (17), and 199 (3.7%) created a premature stop codon. Among these protein-truncating mutations, 58 were located in an essential gene (https://www.yeastgenome.org/) and all were heterozygous, likely phenotypically recessive.

The mutation frequencies per strain genotype (normalized per clone and passage) are reported in Fig. 1*C*. Both BY and SK1/BY wild-type strains accumulated few SNPs, corresponding to a frequency of 0.11 mutations per clone per passage or 1.8 × 10^−10^ mutations per nucleotide per generation, similar to previous measurements (18). Not surprisingly, the vast majority were heterozygous (allelic ratio of ∼0.5) but a few appeared as homozygous (see below). The mutation frequencies and genome rearrangements in the mutant MA lines varied up to 83-fold compared with WT (Fig. 1*C* and Dataset S10) and delineated six classes of mutational profiles. The first class, comprising *cac1 cac3* (chromatin assembly factors), *lig4* (nonhomologous end joining), and *pol32* (polymerase δ replication), accumulated few base substitutions, similar to WT (Fig. 1*C*). The second class, represented by *rad51* (homologous recombination) and *tsa1* (oxidative stress), specifically increased base substitutions (20.5- and 13.6-fold, respectively) but seemingly via different mechanisms (see below). Distinctively, *rad51* more than *tsa1* (2.8 × 10^−2^ and 0.2 × 10^−2^ SVs per clone per passage, respectively) enhanced SVs. All were heterozygous intrachromosomal deletions (Dataset S8). Their length varied between 488 and 59,200 nt and most particularly (11/17 cases) occurred between transposable (Ty/LTR [long terminal repeat]) elements and others between repeated homeologous genes (Dataset S8). This is typical of single-strand annealing events, known to be Rad51-independent (19). The third class is defined by *msh2* (mismatch repair) that exhibited a strong increase of base substitutions (26.4-fold) and small InDels (495-fold) with a slight excess (58.7%) of small InDels over base substitutions, as previously observed (20, 21). Notably, as reported for haploid strains (22, 23), there was an excess (81%) of deletions vs. additions within homopolymer tracts. Among all small variants, complex base substitutions were rare (9/2,824) (Fig. 1*C* and Dataset S10). The fourth class of mutant represented by *clb5* and *sic1* (cell-cycle progression), *mre11* (double-strand break repair), and *tho2* (transcription-coupled recombination) exhibited a slight increase (1.5- to 3.6-fold) of base substitutions but also aneuploidies. The fifth class, defined by *rad27* (lagging-strand replication and base-excision repair), yielded the broadest spectrum of mutational events. It exhibited an increase of base substitutions (8.1-fold increase) including a few complex substitution events (76/1,208), small InDels (63-fold increase), mostly located in homopolymers and microsatellites (518/564) with an excess of insertions vs. deletions (73%) but also SVs represented by 26 large deletions (62 to 23,614 nt), and two small duplications (520 and 542 bp) (Dataset S8) as well as aneuploidies (7.8-fold increase) (Dataset S10). Similar to *rad51*, the SVs in *rad27* reached a spontaneous frequency of 4.3 × 10^−2^ per clone per passage. The deletions involved homeologously repeated regions located in cis but fewer (3/26 in *rad27* instead of 11/17 in *rad51*) involved Ty/LTR elements (Dataset S8). The sixth class of mutational profile is represented by *pif1*, affecting various DNA metabolism functions (https://www.yeastgenome.org/), whose major feature is the rapid and complete loss of mitochondrial DNA (*SI Appendix*, Fig. S2*B*). Further, *pif1∆* MA lines exhibit a slight increase of base substitutions (2.5-fold) (Fig. 1 and Dataset S10), consistent with the two- to threefold increase of spontaneous mutagenesis previously observed in WT cells lacking mitochondrial DNA (*rho0*) (24). Compared with our previous analyses of haploid mutants (23), the mutational spectrum and the overall frequencies of SNPs and small InDels per genome in haploid and diploid cells are similar (*SI Appendix*, Fig. S3), indicating no drastic effect of the ploidy variation.

To more broadly characterize all of the mutational landscapes, we also examined variation of mitochondrial DNA (mtDNA) and ribosomal DNA (rDNA) copy number. It was substantially variable in the WT and mutant parental strains with 0 to 103 mtDNA copies and 42 to 122 rDNA copies. In the MA lines, slight changes of mtDNA and rDNA copy number (∼20 copies) occurred from clone to clone (*SI Appendix*, Fig. S2 *B* and *C*) compared with the parent. *lig4* clones increased and *mre11* clones decreased median copy number of mtDNA (34 and 40 copies, respectively). The study of additional MA lines issued from independent parental strains, preferentially with a variable amount of starting mtDNA, would be required to conclude if this is a mutant-specific effect, as observed in other yeast mutants (25), and determine its impact on mutational profiles. In summary, this set of mutator profiles illustrates a variety of mutator behaviors, leading to a considerable variety of mutational loads and mutational landscapes.

## Mutational Signatures

**Fig. 2.**
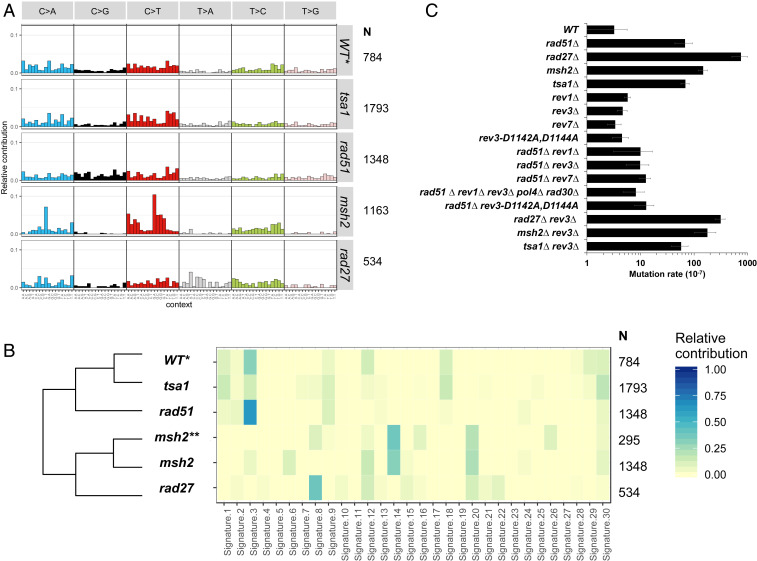
Mutational signatures and Polζ-dependent mutagenesis. (*A*) Mutational profiles of WT, *tsa1*, *rad51*, *msh2*, and *rad27* mutants obtained with MutationalPatterns (63). N: number of base substitutions examined (sum of heterozygous and homozygous SNPs found in BY and SK1/BY backgrounds). Count for *tsa1* is the sum of SNPs observed in the *tsa1∆/∆*, *tsa1∆/∆ pol32∆/∆*, and *tsa1∆/∆ pif1∆/∆* strains (BY and SK1/BY backgrounds). *WT data include our data (110 SNPs) and 719 de novo SNPs detected by Sharp et al. (18) in other WT diploid *S. cerevisiae* MA lines. (*B*) Relative contribution of COSMIC signatures in WT, *tsa1*, *rad51*, *msh2*, and *rad27* mutational profiles (dataset as in *A*), calculated with MutationalPatterns (63). **Data from Lujan et al. (21). (*C*) Canavanine resistance assay of WT and haploid mutants (BY background). The mutation rate is the average of at least three fluctuation tests, each made with five independent cultures. It is calculated according to Reenan and Kolodner (64), using the bz-rates web tool (http://www.lcqb.upmc.fr/bzrates) (65). Error bars are SD.

The landscape of somatic mutations in tumor genomes has been correlated with distinct mutational processes via mathematical and statistical methods able to distinguish different mutational signatures (6, 12, 14, 26–28). It has allowed identification of >30 cancer-derived patterns called COSMIC signatures (https://cancer.sanger.ac.uk/cosmic/signatures) based on the relative incidences of base-substitution changes within a trinucleotide context (12, 26). Similarly, we established the base-substitution profile of our yeast mutants that yielded ≥500 SNP mutations (Fig. 2*A*) and the relative contribution of the COSMIC signatures (Fig. 2*B*). *tsa1*, one of the strongest single-gene mutant mutators in yeast (29), does not exhibit a predominant signature but a near-equal contribution of signatures 1, 3, 9, 18, and 30 (Fig. 2*B*). Thus, loss of Tsa1—the major thioredoxin peroxidase that scavenges hydrogen peroxide in *S. cerevisiae* (30)—which yielded C>A and C>T mutations was not associated with a specific COSMIC signature. Mutations in the human ortholog gene *PRDX1* have not been associated with disease or tumors, perhaps due to extensive functional redundancy of thioredoxin peroxidases in mammals (31). Robustly, the *msh2* signature (C>T, C>A, and T>C) supplemented with homopolymer/microsatellite instability was most similar to signatures 14 and 20 (Fig. 2*B*), consistent with mismatch repair-deficient cancer-derived signatures associated with elevated rates of colorectal and uterine cancers. Our analysis of the *msh2*Δ base substitutions identified by Lujan et al. (21) yielded a similar mutational signature (Fig. 2*B*). By contrast, *rad27* exhibits signature 8 associated with breast cancer and medulloblastoma (https://cancer.sanger.ac.uk/cosmic/signatures). Since *rad27* yields all kinds of mutational events (Fig. 1 *C* and *D*), including a large spectrum of base substitutions (Fig. 2*A*), signature 8 might be the sum of several lesion-specific subsignatures. On the other hand, the *rad51* profile predominantly involving C>A, C>G, and C>T changes exhibited signature 3 (Fig. 2*B*), consistent with its prominent role in homologous recombination (32). Also differently, our analyses of the base substitutions in the mutator DNA polymerase mutants *pol1-L868M*, *pol2-M644G*, and *pol3-L612M* (21) yielded the predominant signatures 8, 22, and 12, respectively (*SI Appendix*, Fig. S4). Altogether, these results outline the uniqueness of the base-substitution signatures to specific genes, and retrospectively inform on the molecular defects underlying the accumulation of mutations in specific tumors (6–15).

### Base Substitution in the Absence of Rad51 Specifically Requires Polζ.

Two decades ago, the elevated mutagenesis of a *rad51* mutant was found to decrease when cells were also mutated in *REV3* (33), a gene now known to encode a component of the error-prone translesion synthesis (TLS) Rev1–Rev3–Rev7 Polζ complex (34). To further explore *rad51* mutagenesis, we associated the *rad51* deletion with each TLS polymerase deletion mutant and measured mutation frequencies with the sensitive *CAN1*^*R*^ mutational assay (35). This revealed that *rad51*-enhanced mutagenesis was reduced essentially to WT levels in combination with *rev1*, *rev3*, or *rev7* but remained unchanged with *pol4* (Polλ) or *rad30* (Polη) (Fig. 2*C*). Consistently, we did not find significant additive or synergic effects of combining *rad51* with the *rev1 rev3 pol4 rad30* quadruple mutant.

Since Rev3 carries the catalytic activity of Polζ while the Rev1 and Rev7 proteins might also serve as “recruitment platforms” involved in other related but distinct biological functions—the mammalian REV7 is involved in controlling DNA end resection and DNA damage responses via the Shieldin complex (36–38)—we also combined *rad51* with the catalytically dead *rev3-D1142A,D144A* polymerase mutant (39). *rad51*-induced mutagenesis was reduced to the WT level (Fig. 2*C*), demonstrating a role for Rev3 TLS activity. Thus, Polζ appeared specifically involved in the default repair of DNA lesions in the absence of Rad51-dependent homologous recombination, most likely during replication. As Polζ is evolutionarily conserved (40), these results raise the possibility that Polζ is responsible for enhanced mutational loads observed in HR-deficient *BRCA1/2* mammalian cells, as well as in patients with *RAD51* mutations and Fanconi anemia-like phenotypes (41).

For comparison, we also combined *rev3* with the other base-substitution mutators. We found no reduction of *CAN1*^*R*^ cells in the *tsa1* background, indicating that Rad52 foci accumulating in this mutant (42) result from a different lesion(s) from in the *rad51* setting. In contrast, the inactivation of *REV3* yielded a partial decrease (58%) of *CAN1*^*R*^ cells when combined with *rad27* (Fig. 2*C*), suggesting that its deficiency in Okazaki fragment processing during lagging-strand replication generates double-strand breaks and/or single-strand gaps similar to *rad51*. The remaining Rev3-independent base-substitution mutations may result from default base-excision repair of apurinic/apyrimidinic sites (43), thus partially contributing to the composite signature 8. Finally, similar to *tsa1*, the lack of Polζ had no discernible effect on *msh2* mutagenesis (Fig. 2*C*). In conclusion, Polζ genetic dependency appears specifically connected to formation and/or resolution of lesions arising in an HR-deficient context.

### Occurrence of Homozygous de Novo Mutations.

**Fig. 3.**
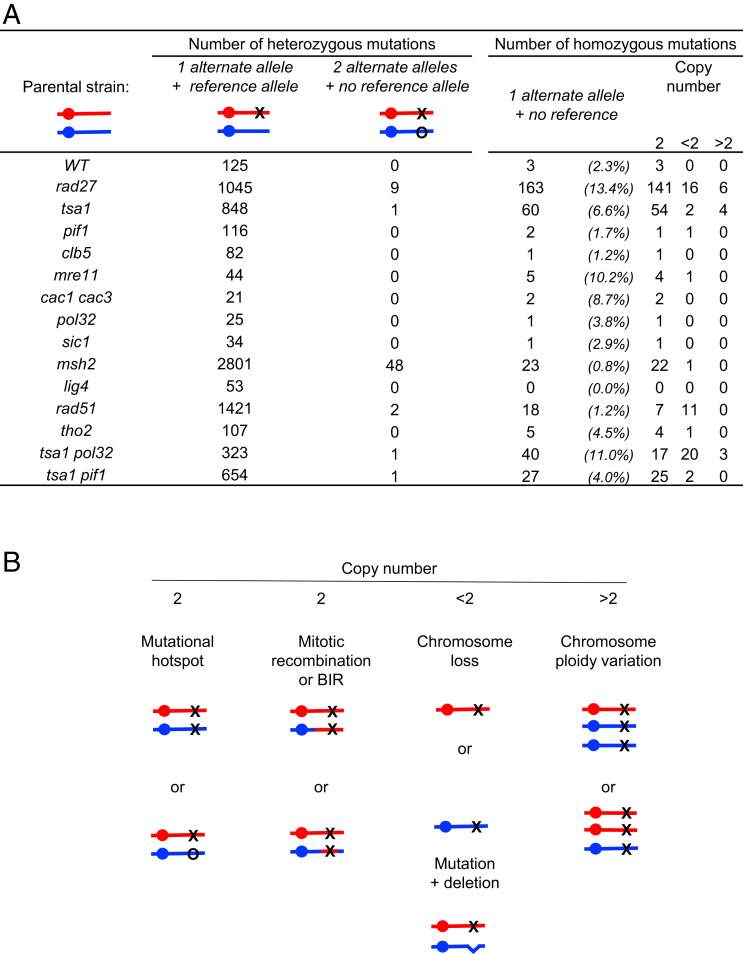
Occurrence and potential origin of “homozygous” de novo mutations. (*A*) Allelic ratio of mutations from MA lines (BY and SK1/BY backgrounds). Sum of heterozygous (allelic ratio ∼0.5) or homozygous (allelic ratio ∼1.0) SNPs, small InDels, MNPs, and complex mutations. Copy number is calculated with Control-FREEC (66). (*B*) Molecular events leading to de novo mutations with an allelic ratio of ∼1.0, associated or not with local or chromosomal copy-number variation.

Beyond heterozygous mutations, we found some base substitutions and InDels with an allelic ratio of 1.0, implying loss of the wild-type allele. This mostly occurred in the *msh2*, *tsa1*, and *rad27* diploids, representing 2.3, 6.6, and 13.4% of the total frequency of base substitutions and small-InDel mutations, respectively (Fig. 3*A* and Dataset S10). In such situations, various types of genomic events, distinguishable by the state of the homologous chromosomes, could be invoked (Fig. 3*B*). In *msh2*, 70/71 cases occurred in full diploid cells and resulted from two identical (18 cases) or two distinct (48 cases) InDels, located within the same homopolymer tract on the homologs (Fig. 3*A* and Datasets S3 and S6). It can be explained from the >1-nt length of these motifs and high rate of polymerase slippage within homopolymers during replication (44). In *tsa1*, the homozygous SNPs were also mostly found on chromosomes with two copies (54/60 cases) but all were located in nonrepeated nucleotide sequences (Fig. 3*A* and Datasets S3 and S4). This was rather similar in *rad27*, except that 22/163 cases were associated with a change of the local copy number (1 or >2). We hypothesize that along the lineages the heterozygous de novo mutations were rendered homozygous upon a subsequent LOH event.

### Detection of LOH Signatures in Hybrid Yeasts.

**Fig. 4.**
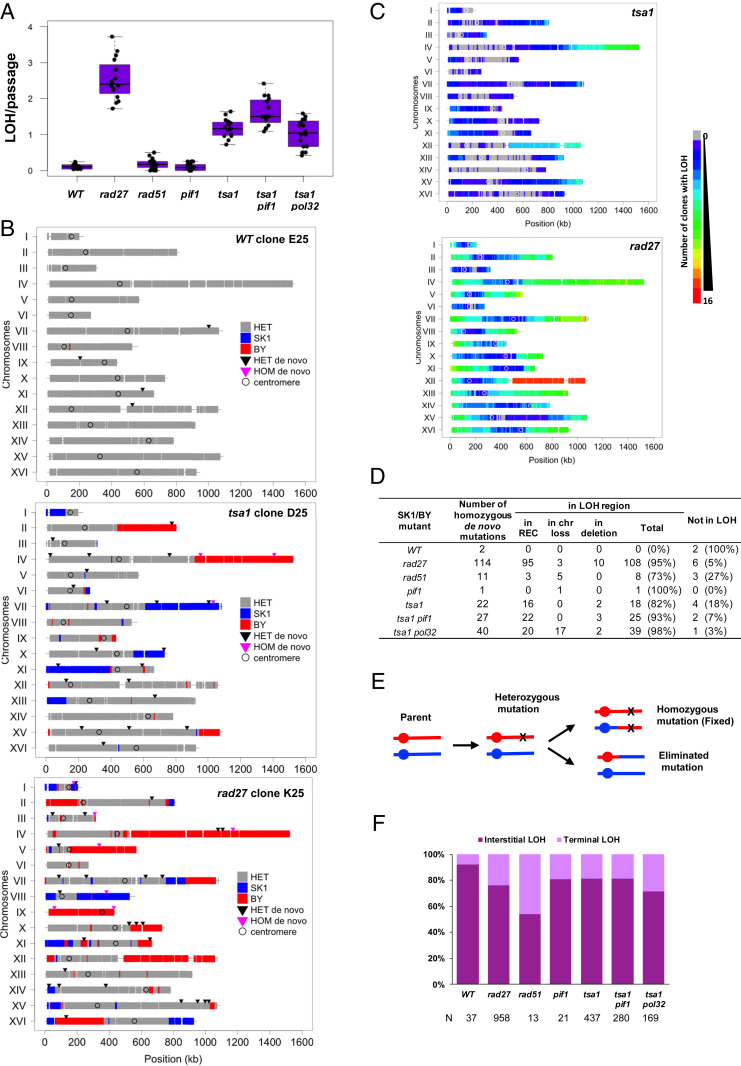
Detection of LOHs in the SK1/BY MA lines. (*A*) Total number of LOH regions per clone normalized per passage. (*B*) Examples of allelic profiles in WT, *tsa1*, and *rad27* MA lines at final passage 25. The genotypes of the 53,523 SK1 vs. BY polymorphisms are plotted on the 16 chromosomes. A minimum of ≥3 adjacent markers of the same parental genotype were retained to define the local haplotype (*SI Appendix*) being either heterozygous SK1/BY (gray), homozygous SK1 (blue), or homozygous BY (red). Triangles indicate the location of heterozygous (black) or homozygous (purple) de novo mutations (SNPs, MNPs, complexes, and small InDels; *SI Appendix*, *Materials and Methods*). (*C*) Heatmaps of the genome-wide occurrence of homozygosity among SK1/BY *tsa1* and *rad27* clones. (*D*) Number of homozygous mutations originated from mitotic recombination or BIR (labeled REC), chromosome loss or deletion identified in the SK1/BY mutants, and being located or not in an LOH region. (*E*) Two-step occurrence of homozygous de novo mutations upon interhomolog mitotic recombination or BIR. (*F*) Percentage of interstitial and terminal LOH–REC tracts. N: total number of LOH events.

LOH can result from mitotic interhomolog recombination, short tract mitotic gene conversions, and/or break-induced replication (BIR) events that are difficult to detect in isogenic strains. To comprehensively detect LOHs, we generated additional WT and mutant MA lines from the polymorphic SK1/BY diploid that carries >53,000 constitutive SNP markers, distributed on each chromosome with one marker every 218 bp on average (Dataset S11). Compared with the isogenic and hybrid WT, the mutant MA lines exhibited similar mutation frequencies and specific mutational landscapes (compare Fig. 1 *C* and *D* and *SI Appendix*, Fig. S2*A*) but revealed the presence of numerous LOH regions, robustly defined to involve ≥3 adjacent markers (Fig. 4*A* and *SI Appendix*, Figs. S5–S9). In WT and *pif1*, LOHs were rare (0.09 and 0.12 LOH per clone per passage, respectively) (*SI Appendix*, Figs. S7 and S8). It was modestly increased in *rad51* (0.18 LOH per clone per passage), while many rose in *tsa1* and *rad27* (1.2 and 2.5 LOHs per clone per passage corresponding to a 12.7- and 27.3-fold increase, respectively). In numerous instances, these LOH events involved several chromosomes in the same clone (Fig. 4*B* and *SI Appendix*, Figs. S5*A* and S6*A*). Considering all of the clones, the LOHs covered a large fraction of the genome in *tsa1* and almost all of the genome in *rad27* (Fig. 4*C*). Regarding the occurrence of homozygous mutations, again this was most frequent in *tsa1* and *rad27* cells (Fig. 4*D* and Dataset S10). Notably, 16/22 in *tsa1* and 95/114 in *rad27* were located in LOH regions with two copies of the chromosome (Fig. 4 *C* and *E* and Datasets S3 and S4), consistent with the hypothesis that along the cell lineage, mutations arose as heterozygous and passively became homozygous as part of a subsequent overlapping LOH event (Fig. 4*E*). Among the remaining events, 2 cases in *tsa1* and 10 cases in *rad27* resulted from the occurrence of a de novo mutation on one homolog and an overlapping de novo deletion on the other homolog (Figs. 3*B* and 4*D* and Dataset S4), as frequently found in tumor cells that carry a germline susceptibility mutation and then acquire a secondary somatic deletion on the homologous chromosome (45). Thus, the highly mutagenic *tsa1* and *rad27* strains stimulated SNPs and LOH events, a dual signature that accelerates and enlarges the spectrum of genome modifications.

### Distributions and Mechanisms of Interstitial and Terminal LOHs.

In *tsa1* and *rad27*, the majority of LOHs were interstitial (81 and 76%, respectively; Fig. 4*F*), with a length varying from 33 bp to 419 kb and 17 bp to 846 kb, respectively (Dataset S12). The remaining LOHs were terminal, with lengths varying from 659 bp to 1,052 kb in *tsa1* and 55 bp to 1,079 kb in *rad27* (Dataset S12). Globally, the interstitial LOHs are shorter than the terminal LOHs (*SI Appendix*, Fig. S10*A*), consistent with their origin resulting from gene conversion-like events and/or double cross-overs rather than a single cross-over. The LOH size ranges were similar to those observed in a previous study (46). In both mutants, the LOHs were from one or the other parental haplotype, with a slight BY vs. SK1 excess genotype (58 and 55%, respectively). Due to the extended polymorphism of the BY and SK1 genomes, this slight bias may result from intrinsic and emerging lethal allele incompatibilities when part of the genome becomes homozygous, a somatic manifestation of the spore inviability observed in the SK1/S288C haploid segregants (47, 48). The annotation of the LOH breakpoint regions did not localize to specific functional elements except in *pif1*, where they often were in proximity to an LTR/Ty region and/or the rDNA locus (*SI Appendix*, Fig. S10*B*). Thus, after only 25 single-bottleneck passages, the stimulation of LOH created mosaic diploid genomes (Fig. 4*C* and *SI Appendix*, Figs. S5*A* and S6*A*) that reached 4.7 to 28.9% homozygosity per clone in *tsa1*, and 26.6 to 60.7% in *rad27*.

The formation of terminal LOHs is a hallmark of BIR (49, 50), whereas both terminal and interstitial LOHs can result from mitotic cross-over recombination and/or gene conversion. Since BIR specifically depends on the activity of *POL32* and *PIF1* (51–54), we examined the effect of deleting these genes in the *tsa1* mutant (Dataset S1). Similar to *tsa1*, the *tsa1 pol32* and *tsa1 pif1* SK1/BY MA lines displayed increased base substitutions (13.9 and 32.3-fold vs. WT, respectively) and LOHs (11.1- and 17.5-fold vs. WT, respectively). The absolute frequency of terminal LOHs, however, was not significantly reduced (0.22, 0.25, and 0.29 per clone per passage in *tsa1*, *tsa1 pol32*, and *tsa1 pif1*, respectively) and the large excess of interstitial vs. terminal LOHs was retained (81, 72, and 81% in *tsa1*, *tsa1 pol32*, and *tsa1 pif1*, respectively) (Fig. 4*F*). Thus, such LOHs result from stimulation of mitotic recombination, rather than BIR, explaining the synthetic lethality of the *tsa1 rad51* double mutant (42). We examined the length of the terminal LOH in *tsa1*, *tsa1 pif1*, and *tsa1 pol32* (*SI Appendix*, Fig. S10*A*) and observed no significant difference between *tsa1* and *tsa1 pif1* but a significant increase of terminal LOH length in *tsa1 pol32*, suggesting a role of Pol32 in the distribution of the initiating events although the annotation of the terminal LOH breakpoints in the three *tsa1* strains is similar (*SI Appendix*, Fig. S10*C*). The contribution of BIR to the stimulation of the *rad27* LOHs could not be examined due to the synthetic lethality of the *rad27 pol32* double mutant (55). Nevertheless, the synthetic lethality of *rad27* (like *tsa1*) with *rad51* (55) suggests that *rad27* LOHs also largely result from interhomolog mitotic recombination, albeit not necessarily stimulated by an identical initiating lesion(s).

### Trajectory of Base Substitution and LOH along Lineages.

**Fig. 5.**
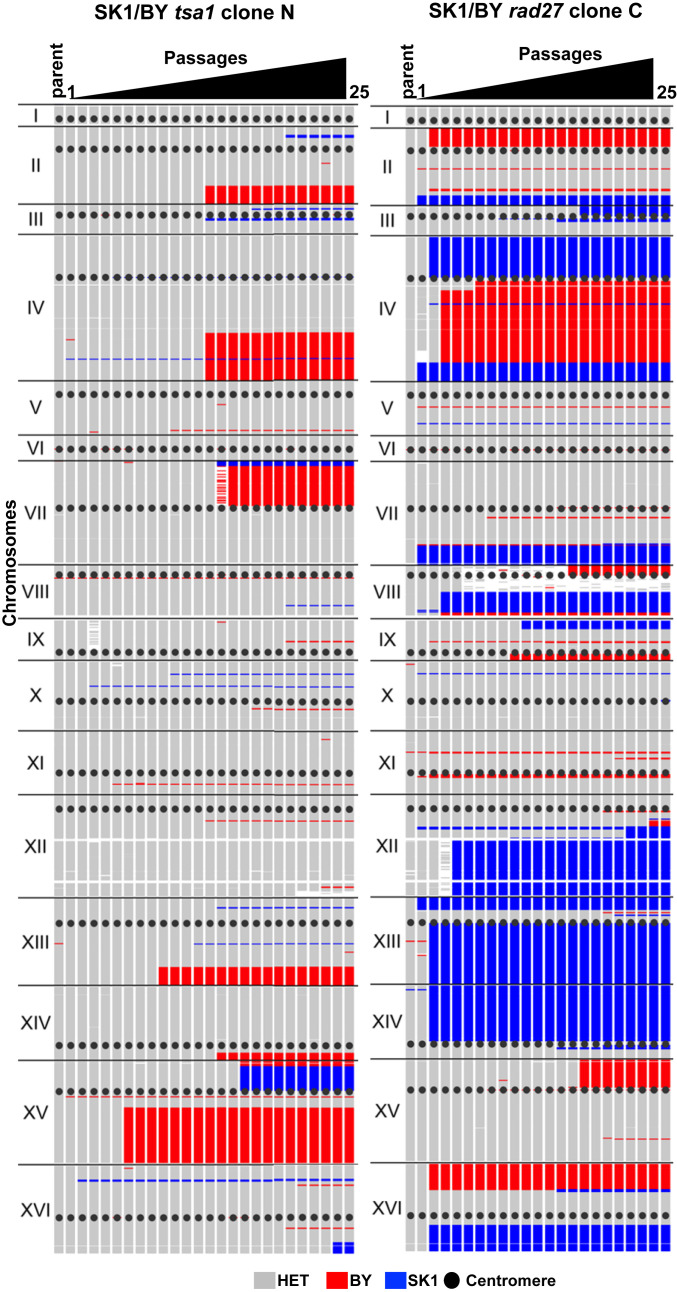
Dynamics of LOH formation in the *tsa1* clone N and *rad27* clone C lineages. Trajectory of the de novo LOH events in SK1/BY *tsa1* clone N (*Left*) and *rad27* clone C (*Right*) from passages 1 to 25. The first column is the parental clone. Gray: SK1/BY heterozygous markers; red: homozygous BY markers; blue: SK1 homozygous markers. In *rad27*, passages 2 and 3 were mosaic and are shown in *SI Appendix*, Fig. S6; passage 8 is omitted because the cells could not be recovered after storage.

To determine trajectories of mutation accumulation, we sequenced the genomes of *tsa1* clone N and *rad27* clone C cells collected at each of the 25 bottleneck passages (*SI Appendix*, Figs. S5*B* and S6*B* and Movies S1 and S2, respectively). In both mutants, the accumulation of heterozygous mutations (SNPs and small InDels) appeared essentially regular; in *tsa1*, 15/25 passages yielded one or two de novo mutations and 7/25 passages yielded three or five mutations; in *rad27*, 7/25 passages yielded one or two mutations but a majority of passages (15/25) yielded three to nine mutations. These multiple mutation events did not necessarily arise within one cell division, since in our experimental protocol, each bottleneck passage corresponds to ∼25 generations (Fig. 5 and Datasets S13 and S14). Also, note, 3/16 *tsa1 pol32* clones (C12, D12, O12) that exhibited an LOH in both the *MSH2* and *PMS1* regions showed a higher number of de novo mutations (34, 30, and 46 mutations, respectively) compared with 19 mutations on average in the other clones. This can be explained by the presence of the *MLH1-D161* homozygous allele from BY and *PMS1-K818* homozygous allele from SK1, previously reported to confer a mismatch repair-deficient phenotype in haploid strains (56). This case illustrates the occurrence of a secondary mutator phenotype occurring during the clonal drift.

**Fig. 6.**
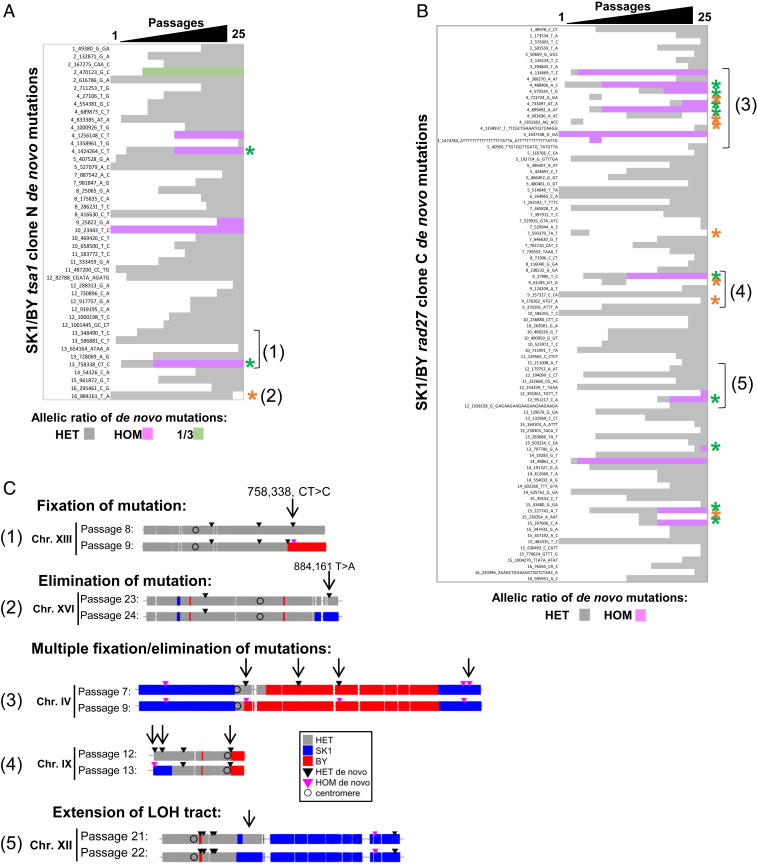
Dynamics of de novo mutations and LOH formation in the *tsa1* clone N and *rad27* clone C lineages. (*A* and *B*) Trajectory and heterozygous (gray) vs. homozygous (purple) status of the 45 de novo mutations detected in *tsa1* clone N and of the 90 de novo mutations detected in *rad27* clone C, respectively, from passages 1 to 25. Green: mutation found in a three–copy-number region and exhibiting a 1/3 allelic ratio. The coordinates of the mutations (chromosome number, position, nucleotides in the parental BY reference, nucleotides in the mutant clone) are shown. Green star: heterozygous mutation that became homozygous; orange star: mutations eliminated in a single passage. Numbers in parentheses refer to chromosomes shown in *C*. (*C*) Examples of fixation and elimination of mutations upon LOH and of mutations associated with the occurrence or extension of a nearby LOH event.

Along the lineages, several de novo heterozygous mutations (2/45 and 9/90 in *tsa1* and *rad27*, respectively) chronologically became homozygous in a single-bottleneck passage as a consequence of an overlapping LOH, while others (1/45 and 6/90 in *tsa1* and *rad27*, respectively) were eliminated in favor of the WT allele (Fig. 6 *A* and *B*, Datasets S15 and S16, and Movies S1 and S2). This opposite outcome is explained by the occurrence of an overlapping LOH mediated by an interhomolog recombination event, followed by the segregation of the nonsister chromatids carrying both WT or mutant alleles in the daughter cells (Fig. 4*E*). Multiple fixations and eliminations of mutations, as well as extensions of LOH tracts, also occurred in a single passage (Fig. 6*C*). The biological impact of such a mutator phenotype is functionally important because during cell proliferation, stimulation of LOHs will allow the phenotypic expression of recessive de novo mutations when fixed but also erase heterozygous mutations that transiently occurred during clonal evolution. In cancer settings, such a mutator phenotype could be initially advantageous to enhance the genetic diversity to stimulate proliferation of pretumoral cells, while afterward the restoration of the WT allele could be beneficial to restore cell physiology. A similar scenario for a dominant mutation in a mutator gene will permit a wave of cell genetic diversification and its subsequent elimination, avoiding the accumulation of additional disadvantageous mutations (2, 57, 58). Retrospectively, in contrast to reversible epigenetic events that may not leave long-term molecular scars, a transient mutation can remain detectable as an LOH event. This “archaeological signature” raises the prospect that one or more LOH-embedded genes may have been transiently mutated during the evolutionary history of a cell lineage.

## Conclusion

The mutation of genes controlling genome stability and/or the epigenetic deregulation of their expression contributes to create the genetic diversity on which Darwinian selection can act. Our study has illustrated the large variety of mutational profiles generated by genetic deficiencies in genome-stability genes and described the dynamics of de novo mutations and genome rearrangements (fixation and disappearance) during vegetative growth. This knowledge suggests ways to mechanistically interpret tumor cell genome evolution and genetic sensitivity (6–11, 59, 60), as well as genome evolution in species (1–3). On the evolutionary scale, impaired function of genes such as *RAD27/FEN1* and *TSA1/PRDX1* may allow the generation of genetic diversity, including occasionally beneficial mutations (or suppressors of less fit mutant states), while additional recombination-dependent changes may be beneficial to resolve burdens of allelic incompatibilities in polymorphic and hybrid species. In the future, extending analyses of mutomes in yeast should allow refinement of the mutator scope of additional genome maintenance genes and graph the complexity of the genes/pathways and their interactions (4). It will also likely suggest how related phenomena operate in other organisms such as *Caenorhabditis elegans* (61) and engineered human cell lines (62) amenable to mutome analyses.

## Materials and Methods

### Strains and Mutation Accumulation Lines.

Mutation accumulation lines were obtained from BY or BY/SK1 diploid mutants carrying homozygous deletions of the genes listed in Fig. 1*A*. The details of the strain constructions are described in *SI Appendix* and the complete strain genotypes are listed in Dataset S1. All strain constructions were checked by PCR, Southern blot, or Sanger sequencing. Proper gene deletions were confirmed by the lack of read coverage upon whole-genome sequencing of the parental and MA lines.

### Generation of Mutation Accumulation Lines.

The mutation accumulation lines were obtained as described in ref. 23. Briefly, 4 to 16 colonies of each diploid parental strain were subjected to 12 to 100 single-cell bottlenecks (Datasets S2 and S10). One single-cell bottleneck is performed by picking one colony of average size and streaking it to obtain individual colonies on a YPD plate (1% yeast extract, 2% peptone, 2% dextrose), incubated for 3 d of growth at 30 °C.

### Mutation Calling, LOH Detection, and Mutational Signatures.

Illumina whole-genome sequencing was performed on parents and mutation accumulation lines. Paired-end reads were aligned on the R64-1-1 S288c *Saccharomyces* Genome Database reference sequence (https://www.yeastgenome.org/). Our analysis pipeline outlined in *SI Appendix*, Fig. S1 allowed the detection of all kinds of mutations and genome rearrangements and ploidy variations. The base-substitution mutational signatures were extracted using the R/Bioconductor MutationalPatterns package (63). In the hybrid strains, the LOH regions were detected by genotyping the 53,523 polymorphisms that distinguish the BY and SK1 strain backgrounds (Dataset S11). The LOHs were robustly defined as regions showing at least three consecutive homozygous markers of the same haplotype (see details in *SI Appendix*).

### Canavanine Mutator Assay.

To measure the rate of mutations in the *CAN1* gene, the occurrence of canavanine-resistant colonies in BY haploids was measured as previously described (64). The fluctuation test assays were performed from five independent cultures. The mutation rate was calculated using the bz-rates (65) tool (http://www.lcqb.upmc.fr/bzrates). Reported mutation rates are the average of at least three experiments.

## Supplementary Material

Supplementary File

Supplementary File

Supplementary File

Supplementary File

Supplementary File

Supplementary File

Supplementary File

Supplementary File

Supplementary File

Supplementary File

Supplementary File

Supplementary File

Supplementary File

Supplementary File

Supplementary File

Supplementary File

Supplementary File

Supplementary File

Supplementary File

## Data Availability

Illumina whole-genome sequencing read (.fastq files) data reported in this work have been deposited in the National Center for Biotechnology Information (NCBI): Sequence Read Archive (PRJNA659115 and ERP123805), under the accession numbers detailed in Dataset S2.

## References

[1] Sniegowski P. D., Gerrish P. J., Lenski R. E., Evolution of high mutation rates in experimental populations of *E. coli*. Nature 387, 703–705 (1997). 9192894 10.1038/42701

[2] Thompson D. A., Desai M. M., Murray A. W., Ploidy controls the success of mutators and nature of mutations during budding yeast evolution. Curr. Biol. 16, 1581–1590 (2006). 16920619 10.1016/j.cub.2006.06.070

[3] Nguyen Ba A. N.., High-resolution lineage tracking reveals travelling wave of adaptation in laboratory yeast. Nature 575, 494–499 (2019). 31723263 10.1038/s41586-019-1749-3 PMC6938260

[4] Myung K., Chen C., Kolodner R. D., Multiple pathways cooperate in the suppression of genome instability in *Saccharomyces cerevisiae*. Nature 411, 1073–1076 (2001). 11429610 10.1038/35082608

[5] Putnam C. D., Kolodner R. D., Pathways and mechanisms that prevent genome instability in *Saccharomyces cerevisiae*. Genetics 206, 1187–1225 (2017). 28684602 10.1534/genetics.112.145805 PMC5500125

[6] Stratton M. R., Campbell P. J., Futreal P. A., The cancer genome. Nature 458, 719–724 (2009). 19360079 10.1038/nature07943 PMC2821689

[7] Martincorena I., Campbell P. J., Somatic mutation in cancer and normal cells. Science 349, 1483–1489 (2015). 26404825 10.1126/science.aab4082

[8] Campbell P. J..; ICGC/TCGA Pan-Cancer Analysis of Whole Genomes Consortium, Pan-cancer analysis of whole genomes. Nature 578, 82–93 (2020). 32025007 10.1038/s41586-020-1969-6 PMC7025898

[9] Pleasance E. D.., A comprehensive catalogue of somatic mutations from a human cancer genome. Nature 463, 191–196 (2010). 20016485 10.1038/nature08658 PMC3145108

[10] Nik-Zainal S..; Breast Cancer Working Group of the International Cancer Genome Consortium, Mutational processes molding the genomes of 21 breast cancers. Cell 149, 979–993 (2012). 22608084 10.1016/j.cell.2012.04.024 PMC3414841

[11] Lawrence M. S.., Mutational heterogeneity in cancer and the search for new cancer-associated genes. Nature 499, 214–218 (2013). 23770567 10.1038/nature12213 PMC3919509

[12] Alexandrov L. B..; PCAWG Mutational Signatures Working Group; PCAWG Consortium, The repertoire of mutational signatures in human cancer. Nature 578, 94–101 (2020). 32025018 10.1038/s41586-020-1943-3 PMC7054213

[13] Pleasance E. D.., A small-cell lung cancer genome with complex signatures of tobacco exposure. Nature 463, 184–190 (2010). 20016488 10.1038/nature08629 PMC2880489

[14] Alexandrov L. B..; Australian Pancreatic Cancer Genome Initiative; ICGC Breast Cancer Consortium; ICGC MMML-Seq Consortium; ICGC PedBrain, Signatures of mutational processes in human cancer. Nature 500, 415–421 (2013). 23945592 10.1038/nature12477 PMC3776390

[15] Kass E. M., Moynahan M. E., Jasin M., When genome maintenance goes badly awry. Mol. Cell 62, 777–787 (2016). 27259208 10.1016/j.molcel.2016.05.021 PMC4966655

[16] Knijnenburg T. A.., Genomic and molecular landscape of DNA damage repair deficiency across The Cancer Genome Atlas. Cell Rep. 23, 239–254.e6 (2018). 29617664 10.1016/j.celrep.2018.03.076 PMC5961503

[17] Cingolani P.., A program for annotating and predicting the effects of single nucleotide polymorphisms, SnpEff: SNPs in the genome of *Drosophila melanogaster* strain w1118; iso-2; iso-3. Fly (Austin) 6, 80–92 (2012). 22728672 10.4161/fly.19695 PMC3679285

[18] Sharp N. P., Sandell L., James C. G., Otto S. P., The genome-wide rate and spectrum of spontaneous mutations differ between haploid and diploid yeast. Proc. Natl. Acad. Sci. U.S.A. 115, E5046–E5055 (2018). 29760081 10.1073/pnas.1801040115 PMC5984525

[19] Ivanov E. L., Sugawara N., Fishman-Lobell J., Haber J. E., Genetic requirements for the single-strand annealing pathway of double-strand break repair in *Saccharomyces cerevisiae*. Genetics 142, 693–704 (1996). 8849880 10.1093/genetics/142.3.693 PMC1207011

[20] Lang G. I., Parsons L., Gammie A. E., Mutation rates, spectra, and genome-wide distribution of spontaneous mutations in mismatch repair deficient yeast. G3 (Bethesda) 3, 1453–1465 (2013). 23821616 10.1534/g3.113.006429 PMC3755907

[21] Lujan S. A.., Heterogeneous polymerase fidelity and mismatch repair bias genome variation and composition. Genome Res. 24, 1751–1764 (2014). 25217194 10.1101/gr.178335.114 PMC4216917

[22] Nishant K. T.., The baker’s yeast diploid genome is remarkably stable in vegetative growth and meiosis. PLoS Genet. 6, e1001109 (2010). 20838597 10.1371/journal.pgen.1001109 PMC2936533

[23] Serero A., Jubin C., Loeillet S., Legoix-Né P., Nicolas A. G., Mutational landscape of yeast mutator strains. Proc. Natl. Acad. Sci. U.S.A. 111, 1897–1902 (2014). 24449905 10.1073/pnas.1314423111 PMC3918763

[24] Rasmussen A. K., Chatterjee A., Rasmussen L. J., Singh K. K., Mitochondria-mediated nuclear mutator phenotype in *Saccharomyces cerevisiae*. Nucleic Acids Res. 31, 3909–3917 (2003). 12853606 10.1093/nar/gkg446 PMC165961

[25] Puddu F.., Genome architecture and stability in the *Saccharomyces cerevisiae* knockout collection. Nature 573, 416–420 (2019). 31511699 10.1038/s41586-019-1549-9 PMC6774800

[26] Alexandrov L. B., Nik-Zainal S., Wedge D. C., Campbell P. J., Stratton M. R., Deciphering signatures of mutational processes operative in human cancer. Cell Rep. 3, 246–259 (2013). 23318258 10.1016/j.celrep.2012.12.008 PMC3588146

[27] Nik-Zainal S., Morganella S., Mutational signatures in breast cancer: The problem at the DNA level. Clin. Cancer Res. 23, 2617–2629 (2017). 28572256 10.1158/1078-0432.CCR-16-2810 PMC5458139

[28] Maura F.., A practical guide for mutational signature analysis in hematological malignancies. Nat. Commun. 10, 2969 (2019). 31278357 10.1038/s41467-019-11037-8 PMC6611883

[29] Huang M.-E., Rio A.-G., Nicolas A., Kolodner R. D., A genomewide screen in *Saccharomyces cerevisiae* for genes that suppress the accumulation of mutations. Proc. Natl. Acad. Sci. U.S.A. 100, 11529–11534 (2003). 12972632 10.1073/pnas.2035018100 PMC208792

[30] Iraqui I.., Peroxiredoxin Tsa1 is the key peroxidase suppressing genome instability and protecting against cell death in *Saccharomyces cerevisiae*. PLoS Genet. 5, e1000524 (2009). 19543365 10.1371/journal.pgen.1000524 PMC2688748

[31] Wood Z. A., Schröder E., Robin Harris J., Poole L. B., Structure, mechanism and regulation of peroxiredoxins. Trends Biochem. Sci. 28, 32–40 (2003). 12517450 10.1016/s0968-0004(02)00003-8

[32] Jensen R. B., Carreira A., Kowalczykowski S. C., Purified human BRCA2 stimulates RAD51-mediated recombination. Nature 467, 678–683 (2010). 20729832 10.1038/nature09399 PMC2952063

[33] Kunz B. A., Ramachandran K., Vonarx E. J., DNA sequence analysis of spontaneous mutagenesis in *Saccharomyces cerevisiae*. Genetics 148, 1491–1505 (1998). 9560369 10.1093/genetics/148.4.1491 PMC1460101

[34] Nelson J. R., Lawrence C. W., Hinkle D. C., Thymine-thymine dimer bypass by yeast DNA polymerase ζ. Science 272, 1646–1649 (1996). 8658138 10.1126/science.272.5268.1646

[35] Lang G. I., Murray A. W., Estimating the per-base-pair mutation rate in the yeast *Saccharomyces cerevisiae*. Genetics 178, 67–82 (2008). 18202359 10.1534/genetics.107.071506 PMC2206112

[36] Gupta R.., DNA repair network analysis reveals Shieldin as a key regulator of NHEJ and PARP inhibitor sensitivity. Cell 173, 972–988.e23 (2018). 29656893 10.1016/j.cell.2018.03.050 PMC8108093

[37] Tomida J.., FAM35A associates with REV7 and modulates DNA damage responses of normal and BRCA1-defective cells. EMBO J. 37, e99543 (2018). 29789392 10.15252/embj.201899543 PMC6003645

[38] Dev H.., Shieldin complex promotes DNA end-joining and counters homologous recombination in BRCA1-null cells. Nat. Cell Biol. 20, 954–965 (2018). 30022119 10.1038/s41556-018-0140-1 PMC6145444

[39] Siebler H. M., Lada A. G., Baranovskiy A. G., Tahirov T. H., Pavlov Y. I., A novel variant of DNA polymerase ζ, Rev3ΔC, highlights differential regulation of Pol32 as a subunit of polymerase δ versus ζ in *Saccharomyces cerevisiae*. DNA Repair (Amst.) 24, 138–149 (2014). 24819597 10.1016/j.dnarep.2014.04.013 PMC4225194

[40] Gan G. N., Wittschieben J. P., Wittschieben B. Ø., Wood R. D., DNA polymerase zeta (pol zeta) in higher eukaryotes. Cell Res. 18, 174–183 (2008). 18157155 10.1038/cr.2007.117

[41] Wang A. T.., A dominant mutation in human RAD51 reveals its function in DNA interstrand crosslink repair independent of homologous recombination. Mol. Cell 59, 478–490 (2015). 26253028 10.1016/j.molcel.2015.07.009 PMC4529964

[42] Ragu S.., Oxygen metabolism and reactive oxygen species cause chromosomal rearrangements and cell death. Proc. Natl. Acad. Sci. U.S.A. 104, 9747–9752 (2007). 17535927 10.1073/pnas.0703192104 PMC1887571

[43] Wu X., Wang Z., Relationships between yeast Rad27 and Apn1 in response to apurinic/apyrimidinic (AP) sites in DNA. Nucleic Acids Res. 27, 956–962 (1999). 9927726 10.1093/nar/27.4.956 PMC148273

[44] Strand M., Prolla T. A., Liskay R. M., Petes T. D., Destabilization of tracts of simple repetitive DNA in yeast by mutations affecting DNA mismatch repair. Nature 365, 274–276 (1993). 8371783 10.1038/365274a0

[45] Li Y..; PCAWG Structural Variation Working Group; PCAWG Consortium, Patterns of somatic structural variation in human cancer genomes. Nature 578, 112–121 (2020). 32025012 10.1038/s41586-019-1913-9 PMC7025897

[46] Yim E., O’Connell K. E., St Charles J., Petes T. D., High-resolution mapping of two types of spontaneous mitotic gene conversion events in *Saccharomyces cerevisiae*. Genetics 198, 181–192 (2014). 24990991 10.1534/genetics.114.167395 PMC4174931

[47] Laureau R.., Extensive recombination of a yeast diploid hybrid through meiotic reversion. PLoS Genet. 12, e1005781 (2016). 26828862 10.1371/journal.pgen.1005781 PMC4734685

[48] Martini E., Diaz R. L., Hunter N., Keeney S., Crossover homeostasis in yeast meiosis. Cell 126, 285–295 (2006). 16873061 10.1016/j.cell.2006.05.044 PMC1949389

[49] Llorente B., Smith C. E., Symington L. S., Break-induced replication: What is it and what is it for? Cell Cycle 7, 859–864 (2008). 18414031 10.4161/cc.7.7.5613

[50] Malkova A., Ira G., Break-induced replication: Functions and molecular mechanism. Curr. Opin. Genet. Dev. 23, 271–279 (2013). 23790415 10.1016/j.gde.2013.05.007 PMC3915057

[51] Lydeard J. R., Jain S., Yamaguchi M., Haber J. E., Break-induced replication and telomerase-independent telomere maintenance require Pol32. Nature 448, 820–823 (2007). 17671506 10.1038/nature06047

[52] Saini N.., Migrating bubble during break-induced replication drives conservative DNA synthesis. Nature 502, 389–392 (2013). 24025772 10.1038/nature12584 PMC3804423

[53] Wilson M. A.., Pif1 helicase and Polδ promote recombination-coupled DNA synthesis via bubble migration. Nature 502, 393–396 (2013). 24025768 10.1038/nature12585 PMC3915060

[54] Donnianni R. A.., DNA polymerase delta synthesizes both strands during break-induced replication. Mol. Cell 76, 371–381.e4 (2019). 31495565 10.1016/j.molcel.2019.07.033 PMC6862718

[55] Loeillet S.., Genetic network interactions among replication, repair and nuclear pore deficiencies in yeast. DNA Repair (Amst.) 4, 459–468 (2005). 15725626 10.1016/j.dnarep.2004.11.010

[56] Demogines A., Wong A., Aquadro C., Alani E., Incompatibilities involving yeast mismatch repair genes: A role for genetic modifiers and implications for disease penetrance and variation in genomic mutation rates. PLoS Genet. 4, e1000103 (2008). 18566663 10.1371/journal.pgen.1000103 PMC2413424

[57] Couce A.., Mutator genomes decay, despite sustained fitness gains, in a long-term experiment with bacteria. Proc. Natl. Acad. Sci. U.S.A. 114, E9026–E9035 (2017). 29073099 10.1073/pnas.1705887114 PMC5664506

[58] Giraud A.., Costs and benefits of high mutation rates: Adaptive evolution of bacteria in the mouse gut. Science 291, 2606–2608 (2001). 11283373 10.1126/science.1056421

[59] Guo E.., FEN1 endonuclease as a therapeutic target for human cancers with defects in homologous recombination. Proc. Natl. Acad. Sci. U.S.A. 117, 19415–19424 (2020). 32719125 10.1073/pnas.2009237117 PMC7431096

[60] Farmer H.., Targeting the DNA repair defect in BRCA mutant cells as a therapeutic strategy. Nature 434, 917–921 (2005). 15829967 10.1038/nature03445

[61] Meier B.., Mutational signatures of DNA mismatch repair deficiency in *C. elegans* and human cancers. Genome Res. 28, 666–675 (2018). 29636374 10.1101/gr.226845.117 PMC5932607

[62] Zou X.., Validating the concept of mutational signatures with isogenic cell models. Nat. Commun. 9, 1744 (2018). 29717121 10.1038/s41467-018-04052-8 PMC5931590

[63] Blokzijl F., Janssen R., van Boxtel R., Cuppen E., MutationalPatterns: Comprehensive genome-wide analysis of mutational processes. Genome Med. 10, 33 (2018). 29695279 10.1186/s13073-018-0539-0 PMC5922316

[64] Reenan R. A. G., Kolodner R. D., Characterization of insertion mutations in the *Saccharomyces cerevisiae* MSH1 and MSH2 genes: Evidence for separate mitochondrial and nuclear functions. Genetics 132, 975–985 (1992). 1334021 10.1093/genetics/132.4.975 PMC1205253

[65] Gillet-Markowska A., Louvel G., Fischer G., bz-rates: A web tool to estimate mutation rates from fluctuation analysis. G3 (Bethesda) 5, 2323–2327 (2015). 26338660 10.1534/g3.115.019836 PMC4632052

[66] Boeva V.., Control-FREEC: A tool for assessing copy number and allelic content using next-generation sequencing data. Bioinformatics 28, 423–425 (2012). 22155870 10.1093/bioinformatics/btr670 PMC3268243

