# Supplementary material for: Trajectory and uniqueness of mutational signatures in yeast mutators
Source: Proc Natl Acad Sci U S A. 2020 Sep 23;117(40):24947–56. doi: 10.1073/pnas.2011332117 (PMC7547211; doi:10.1073/pnas.2011332117)
Supplement: Supplementary File [file pnas.2011332117.sapp.pdf]

Supplementary Information for

Trajectory and Uniqueness of Mutational Signatures in Yeast Mutators

Sophie Loeillet[a,b], Mareike Herzog[c], Fabio Puddu[c], Patricia Legoix[d], Sylvain Baulande[d], Stephen P. Jackson[c] & Alain Nicolas[a,b,*]

[*] Correspondence: alain.nicolas@curie.fr

This PDF file includes:

Supplementary Materials and Methods

Figures S1 to S10

Legends for Movies S1 to S2

Legends for Datasets S1 to S16

SI References

Other supplementary materials for this manuscript include the following:

Movies S1 to S2

Datasets S1 to S16

www.pnas.org/cgi/doi/10.1073/pnas.2011332117

Supplementary Information Text

Materials and Methods

Strains. The strains and their genotypes are listed in Dataset S1. The BY strains are derivatives of BY4741 and BY4742 (1). AND2210 (BY WT) resulted from the mating of BY4741 and BY4742. The BY diploids were generated by crossing the BY *MATa* mutant strains from our previous work (2) with the BY *MATα* corresponding mutants obtained by mating-type switching, sporulation of heterozygous diploids or transformation. The *sic1*Δ, *rad51*Δ, *lig4*Δ haploid strains were obtained by transformation of BY4741 with the *KanMX4* deletion cassettes generated by PCR of the *S. cerevisiae* single-gene deletant collection (3). They were subsequently crossed with the *MATα* corresponding mutant, generated as described above, to form the *sic1*Δ*/sic1*Δ, *rad51*Δ/Δ and *lig4*Δ/Δ diploids. The BY *pol32*Δ/Δ and WT (AND3148) strains were obtained by crossing appropriate spores originated from sporulation of *pol32*Δ/+ *sic1*Δ/+ diploid. BY *tho2*Δ/Δ (ORT6883) arose spontaneously by genome duplication upon transformation of BY4741 with the *tho2::KanMX* deletion cassette. The SK1 strains are derivatives of ORT7281 (4), ORT7237 (4) and ORD7339 (5). SK1 haploid mutants were obtained by transformation with deletion cassettes generated by PCR from our BY *MATa* mutants (2) or from the *S. cerevisiae* single-gene deletant collection (3) then crossed with our BY mutants to form the SK1/BY hybrid mutant diploids. The SK1/BY *rad27*Δ/Δ strain is derivative of the SK1 *rad27*Δ ORD5902-1C (6). AND3189 (SK1/BY WT) is derivative of BY4741 (1) and ORD7339 (5). All strain constructions were checked by PCR or Southern blot. Proper gene deletions were confirmed by the lack of read coverage upon whole genome sequencing. The *rev3-D1142A/D1144A* strain was obtained by transformation of the BY4741 *rev3::URA3* strain using PCR DNA fragment containing the *REV3* D1142A and D1144A mutations and selection of 5-FOA resistant colonies. Full ORF sequence of *REV3* was checked

by Sanger sequencing. *RAD51* was subsequently deleted in this strain to form the *rad51*Δ *rev3-D1142A/D1144A* double-mutant.

Whole genome sequencing and reads mapping. The genomic DNA of the parental strains and mutation accumulation lines was extracted to prepare 2x100 or 150 bp Illumina Paired-End libraries according to manufacturer's protocols. Reads were mapped on R64-1-1 S288c *S. cerevisiae* reference genome release (S288C_reference_genome_R64-1-1_20110203, http://www.yeastgenome.org) using BWA (7). PCR duplicates were removed with Picard (http://broadinstitute.github.io/picard/) prior to mutations calling and depth of coverage calculation.

Mutations calling. The detection of *de novo* mutations was made as described previously (8). Bioinformatic tools were run into graphical interface of Institut Curie's Galaxy (9) instance (http://galaxy-public.curie.fr). Freebayes (10) was used for SNP (Single Nucleotide Polymorphism), MNP (Multiple Nucleotide Polymorphism), "complex" events (combinations of SNP and indels) and small indels detection. g-deNoise (11) filtering was applied to Freebayes mutations lists to exclude calls located into repeated regions; U-genome (unique) size is 11,060,020 bp and is 76,5% coding (8,466,395 positions of this genome is located in coding sequence). For each strain, Freebayes *de novo* mutations were defined as mutations that were (i) absent from the parental strain (for the SK1/BY diploids, all WT BY4741 and SK1 ORT7237 Freebayes calls were also excluded), (ii) specific of one mutation accumulation line, (iii) called with good quality (QUAL>30) and sufficient reads depth (at least ~15% of sample mean depth coverage, see Dataset S2) and, (iv) characterized by an allelic ratio >0.4 and <0.6 (heterozygous calls) or >0.9 (homozygous calls). Positions with 2 alleles calls (NUMALT=2) were counted separately (Dataset S6) and were defined as the mutations where (i) each of the 2 calls were

absent from the parental clone, (ii) both calls were heterozygous (allelic ratio >0.4 and <0.6), (iii) the sum of the 2 calls had an allelic ratio >0.9, (iv) QUAL>30 and, (v) a depth of coverage above threshold (see above). The mutations occurring in more than one clone but not all are reported in Dataset S5, all with QUAL>30 and mean depth coverage above threshold (see above). The potential mutations occurring in the mitochondrial DNA were not considered. The small variants were annotated by means of SnpEff (12). The depth of coverage and copy number variations were calculated with GATK (13) and Control-FREEC (14), respectively. The structural variants (SV) were detected with Lumpy (15) and Delly (16). .vcf files were merged with SURVIVOR (17) (same type, same strand orientation, 175 bp maximum distance parameters). Calls specific of one clone with both callers and reported with at least 10 supporting reads (SU) were kept. Small deletions (<57 nt) detected as indels with Freebayes and the SV pipelines were assigned to the Indel category. To confirm deletions and duplications, ratio between mean depth coverage of the SV region and the genome was calculated and compared to parent. SV with sample/parent ratio <0.78 (see SK1/BY *rad27*Δ/Δ M25 clone deletion on chr. XII, robustly confirmed by LOH) were counted as deletions. SV with sample/parent ratio >1.3 were counted as duplications. Deletions in the SK1/BY hybrids were further confirmed by presence of homozygous polymorphic markers. Mutations lists were processed with custom R scripts.

Mutational signatures. The R/Bioconductor MutationalPatterns (18) package was used to extract *de novo* mutational signatures with non-negative matrix factorization and to compare extracted signatures with COSMIC Signatures (https://cancer.sanger.ac.uk/cosmic/signatures_v2).

rDNA copy number calculation. rDNA copy number was obtained by dividing mean depth coverage of the rDNA locus (chrXII, 451,575-468,931) by the 16 chromosomes mean depth coverage, and multiplying by 2 because the S288C reference genome contains two copies of the minimal repeat unit, as described in (19).

mtDNA copy number calculation. The mitochondrial DNA copy number was obtained as described in (19) by dividing mean depth coverage of a region of the *COX1* gene (14,000-20,000) by the genome mean depth coverage (calculated as the mean of the depth coverage of each 16 chromosomes), and multiplying by 2 as clones are diploid.

***de novo* mutations calling in lineages.** The *de novo* mutations were identified as described above but without any initial filtering on allelic ratio. Only calls found in at least 2 consecutive passages were kept. Calls with allelic ratio ~1/3 or 2/3 in all passages without evidence of gain of coverage were discarded. Allelic ratio of flanking markers were examined for calls with ambiguous allelic ratio along the passages to confirm the heterozygous or homozygous nature of the *de novo* mutations. Altogether, we eliminated 4 and 20 potential mutations in the *tsa1* and *rad27* lineages, respectively. The SK1/BY *rad27* clone C lineage lacks data of passage 8 because no growing cells were recovered from the plates kept at +4°C. The passages 2 and 3 showed unresolved LOH profiles compared with the whole lineage although some *de novo* mutations and LOH showed consistency with the following passages.

SK1 markers genotyping. The SK1 markers list was defined from Illumina whole genome sequencing of the SK1/S288c diploid AND1702 (4) (mean depth coverage = 85X) and variants calling method with Freebayes (heterozygous, QUAL>30, depth of coverage>15 and g-deNoise filtering (11)) using the S288c sequence as reference genome. This list was compared with SK1

haploid (ORT7237 (4)) homozygous variant calls and BY4741 variant calls, to keep the 53,523 SNPs common to SK1 but absent from BY4741 (Dataset S11). Genotyping of the hybrid diploid clones was made by examining the mutation calls at those 53,523 polymorphic positions of the genome, after reads mapping on S288c reference genome. A given position is genotyped as "SK1" if the allelic ratio is >0.9; or "BY" if the allelic ratio is 0 or "heterozygous" if the allelic ratio is between 0.4 and 0.6. Low quality calls (QUAL<30) or with low depth of coverage (lower than 15% mean depth coverage of the sample) were discarded from the genotyping analysis.

***de novo* LOH analysis**. Markers genotyping was made as described above for each mutation accumulation lines. Positions that were scored as homozygous in parental strain were discarded. The LOH regions were robustly defined to include at least 3 consecutives homozygous SK1 markers that exhibited the same allele, high variant calling quality (QUAL>30) and a minimum of ~15% mean depth coverage. The heterozygous regions with ~0.5 allelic ratio (>0.4 and <0.6), the regions with allelic ratio ~1/3 (>0 and ≤0.4) and the regions with allelic ratio ~2/3 (≥0.6 and ≤0.9) were also identified by 3 consecutive markers. Consecutive LOH regions carrying the same parental alleles were merged. The LOH present in all clones of one parent were eliminated. The LOH were considered to be terminal when the first or last homozygous markers were respectively the first or the last genotyped marker of a chromosome, interstitial if not. Copy number of LOH was calculated using Control-FREEC(14) and is the copy number at the middle position of the LOH. LOH junctions were defined as the regions between first/last homozygous SK1 polymorphic marker of the LOH and adjacent non homozygous marker. Annotations were made using SGD R64-1-1 S288c *S. cerevisiae* reference genome (http://www.yeastgenome.org); non annotated regions were reported as "non coding".

Data availability. The NGS data and materials are available upon request.

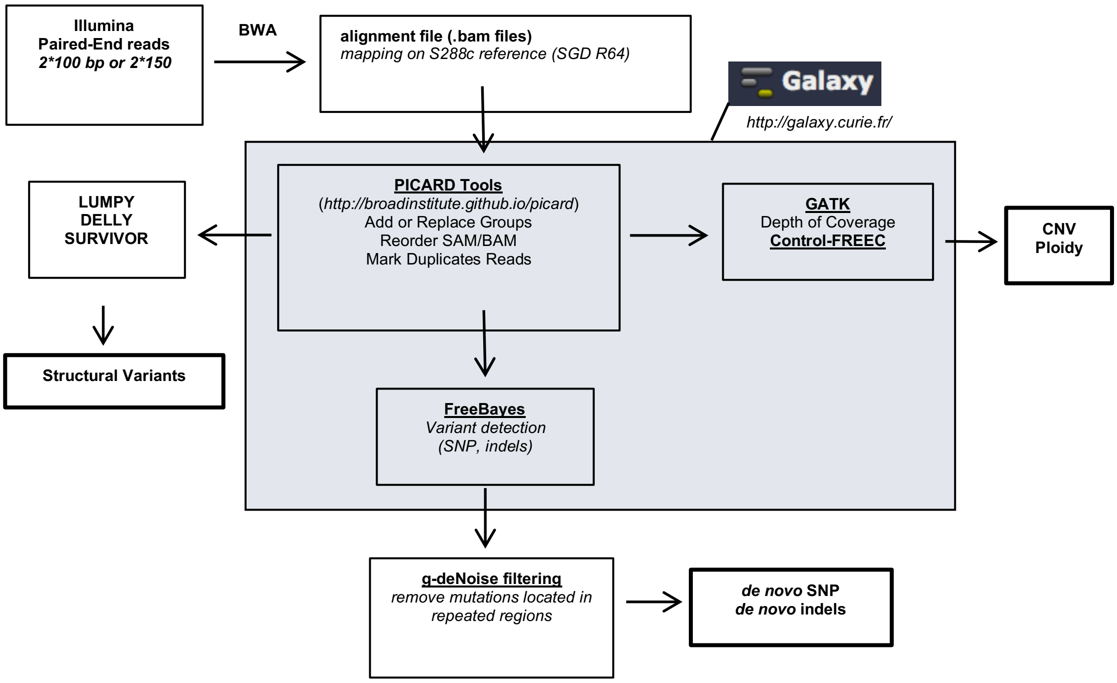

Fig. S1. Bioinformatic analysis workflow of the MA lines. Bioinformatic tools used in this study (see Materials and Methods).

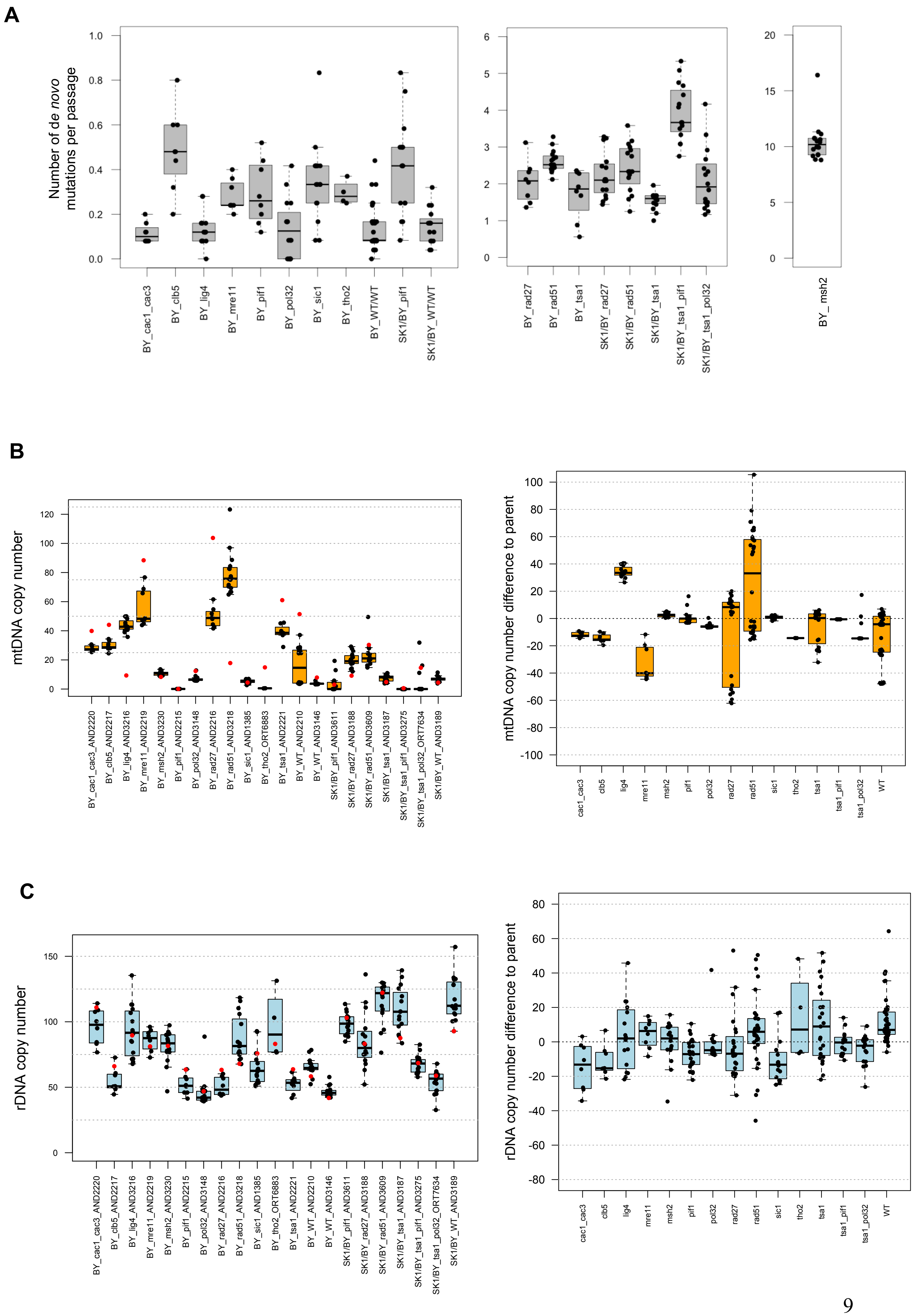
A
Number of de novo mutations per passage
BY_cac1_cac3
BY_clb5
BY_lig4
BY_mre11
BY_pif1
BY_pol32
BY_sic1
BY_tho2
BY_WT/WT
SK1/BY_pif1
SK1/BY_WT/WT
BY_rad27
BY_rad51
BY_tsa1
SK1/BY_rad27
SK1/BY_rad51
SK1/BY_tsa1
SK1/BY_tsa1_pif1
SK1/BY_tsa1_pol32
BY_msh2
B
mtDNA copy number
mtDNA copy number difference to parent
C
rDNA copy number
rDNA copy number difference to parent
BY_cac1_cac3_AND2220
BY_clb5_AND2217
BY_lig4_AND3216
BY_mre11_AND2219
BY_msh2_AND3230
BY_pif1_AND2215
BY_pol32_AND3148
BY_rad27_AND2216
BY_rad51_AND3218
BY_sic1_AND1385
BY_tho2_ORT6883
BY_tsa1_AND2221
BY_WT_AND2210
BY_WT_AND3146
SK1/BY_pif1_AND3611
SK1/BY_rad27_AND3188
SK1/BY_rad51_AND3609
SK1/BY_tsa1_AND3187
SK1/BY_tsa1_pif1_AND3275
SK1/BY_tsa1_pol32_ORT7634
SK1/BY_WT_AND3189
cac1_cac3
clb5
lig4
mre11
msh2
pif1
pol32
rad27
rad51
sic1
tho2
tsa1
tsa1_pif1
tsa1_pol32
WT

Fig. S2. Number of *de novo* mutations per clones. (*A*) Number of *de novo* mutations per passage. Counts are the sum of (i) heterozygous or homozygous SNP, small indels, MNP and complex mutations (Datasets S3 and 4) specific of one clone, (ii) heterozygous or homozygous SNP, small indels, MNP and complex mutations occurring in more than one clone (Dataset S5) or (iii) corresponding to positions with 2 calls (Dataset S6), (iv) number of lost or gained chromosomes (Dataset S7) and (v) structural variants (Dataset S8). Counts are normalized by the number of passages. Left, central and right panels show low (~ 0 to 1 mutation per clone and per passage), medium (~ 1 to 5 mutations per clone and per passage) and high (~ >10 mutations per clone and per passage) mutators, respectively. (*B*) Variations of mtDNA copy number: absolute number of mtDNA copies (left) and loss or gain upon passages (right panel); the parent is shown in red. (*C*) Variations of rDNA copy number: absolute number of rDNA copies (left panel) and loss or gain (right) upon passages. The parent is shown in red.

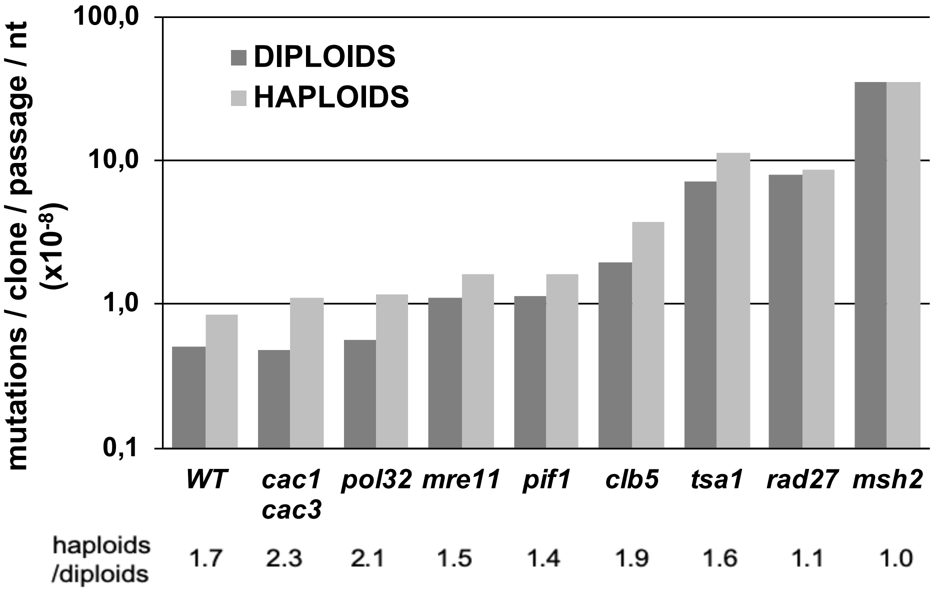

Fig. S3. Number of mutations in haploid and diploid mutations lines (BY background). For haploids, mutations counts are sum of SNP, small indels, aneuploidies and SV from Serero et al (2). For diploids, mutations counts are as in Fig. 1*C* but MNP+complex mutations were not included because they were not scored in haploids. Data were normalized to number of clones, passages and nucleotides.

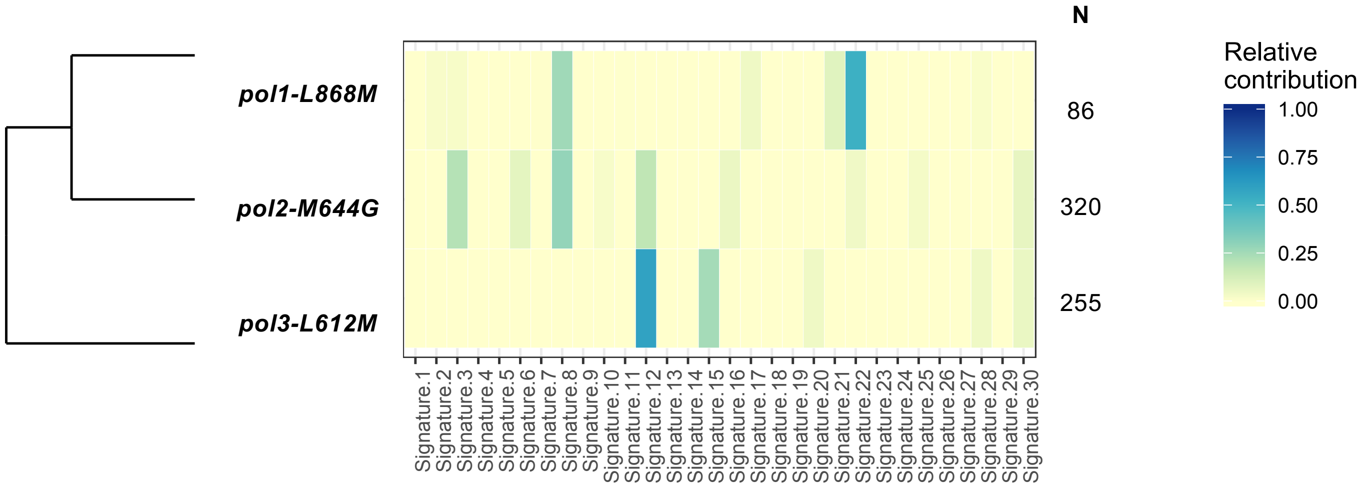

Fig. S4. Relative contribution of COSMIC signatures in *pol1-L868M*, *pol2-M644G* and *pol3-L612M* mutational profiles, calculated with MutationalPatterns (18). Mutation accumulation lines were generated in Lujan et *al*. (20). N: number of bases substitutions examined.

Figure S5

A

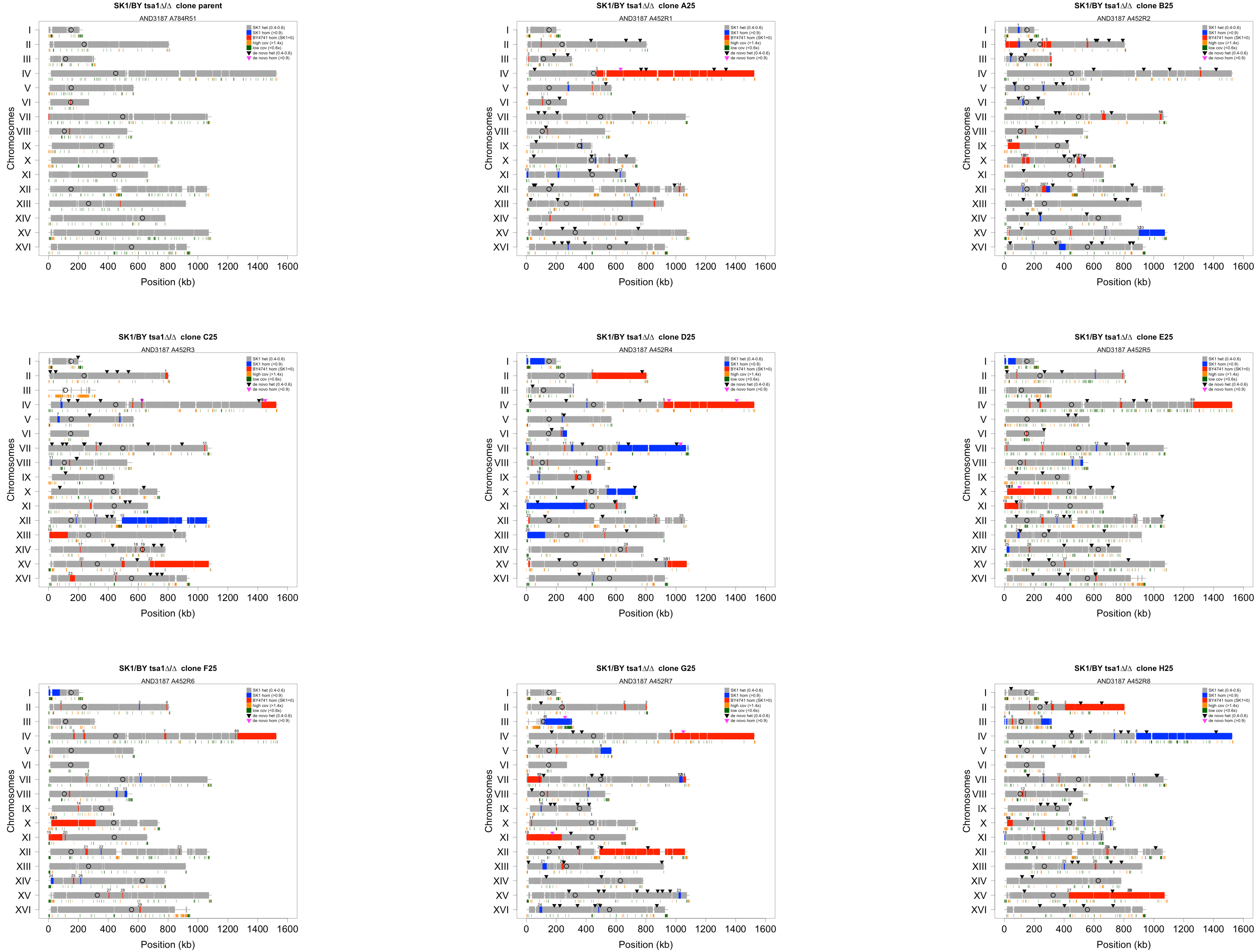
SK1/BY tsa1Δ/Δ clone parent
AND3187 A784R51
SK1/BY tsa1Δ/Δ clone A25
AND3187 A452R1
SK1/BY tsa1Δ/Δ clone B25
AND3187 A452R2
SK1/BY tsa1Δ/Δ clone C25
AND3187 A452R3
SK1/BY tsa1Δ/Δ clone D25
AND3187 A452R4
SK1/BY tsa1Δ/Δ clone E25
AND3187 A452R5
SK1/BY tsa1Δ/Δ clone F25
AND3187 A452R6
SK1/BY tsa1Δ/Δ clone G25
AND3187 A452R7
SK1/BY tsa1Δ/Δ clone H25
AND3187 A452R8
SK1 het (0.4-0.6)
SK1 hom (>0.9)
BY4741 hom (SK1=0)
high cov (>1.4x)
low cov (<0.6x)
de novo het (0.4-0.6)
de novo hom (>0.9)
Chromosomes
I
II
III
IV
V
VI
VII
VIII
IX
X
XI
XII
XIII
XIV
XV
XVI
0 200 400 600 800 1000 1200 1400 1600
Position (kb)

Figure S5 (continue)

A

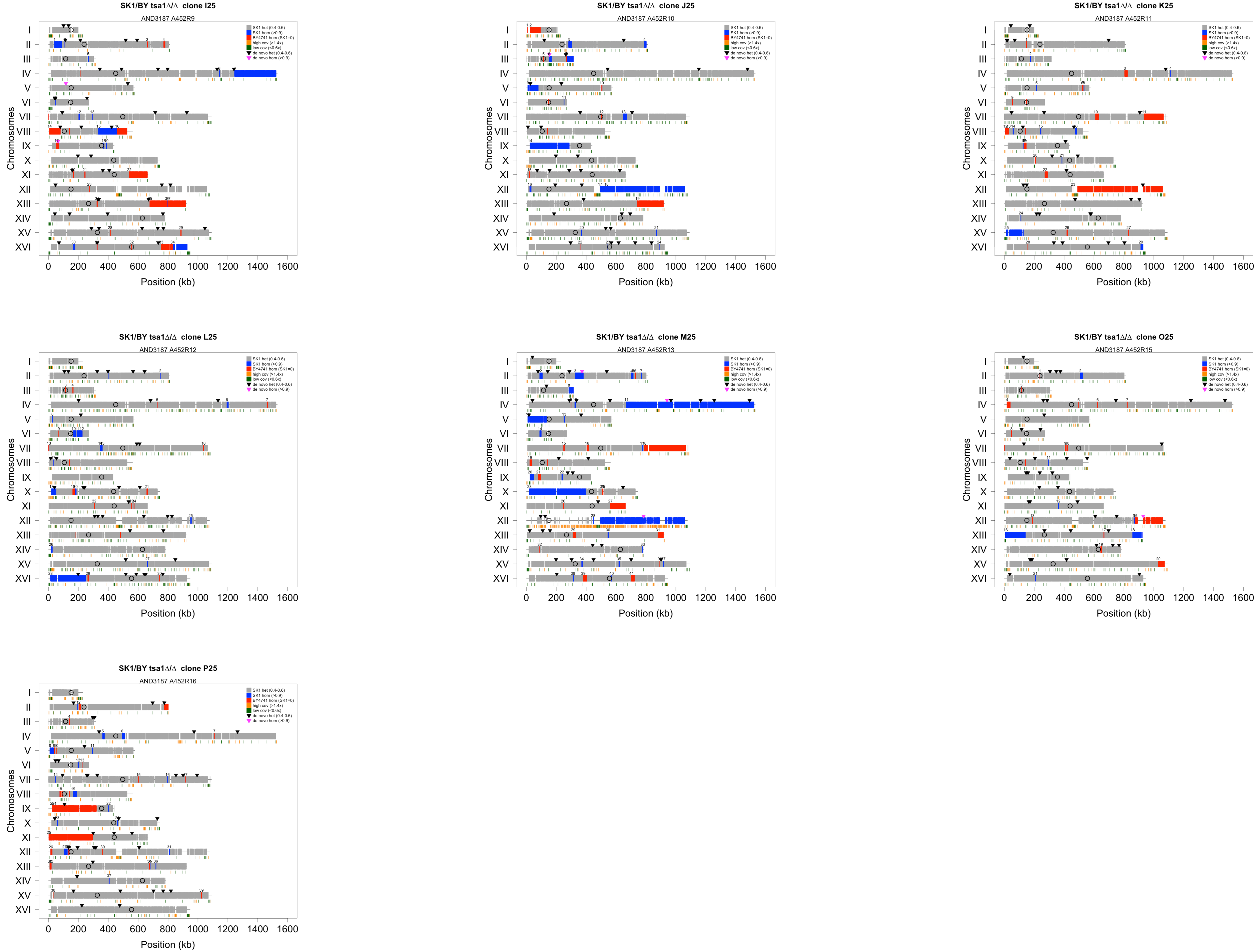

Figure S5

B

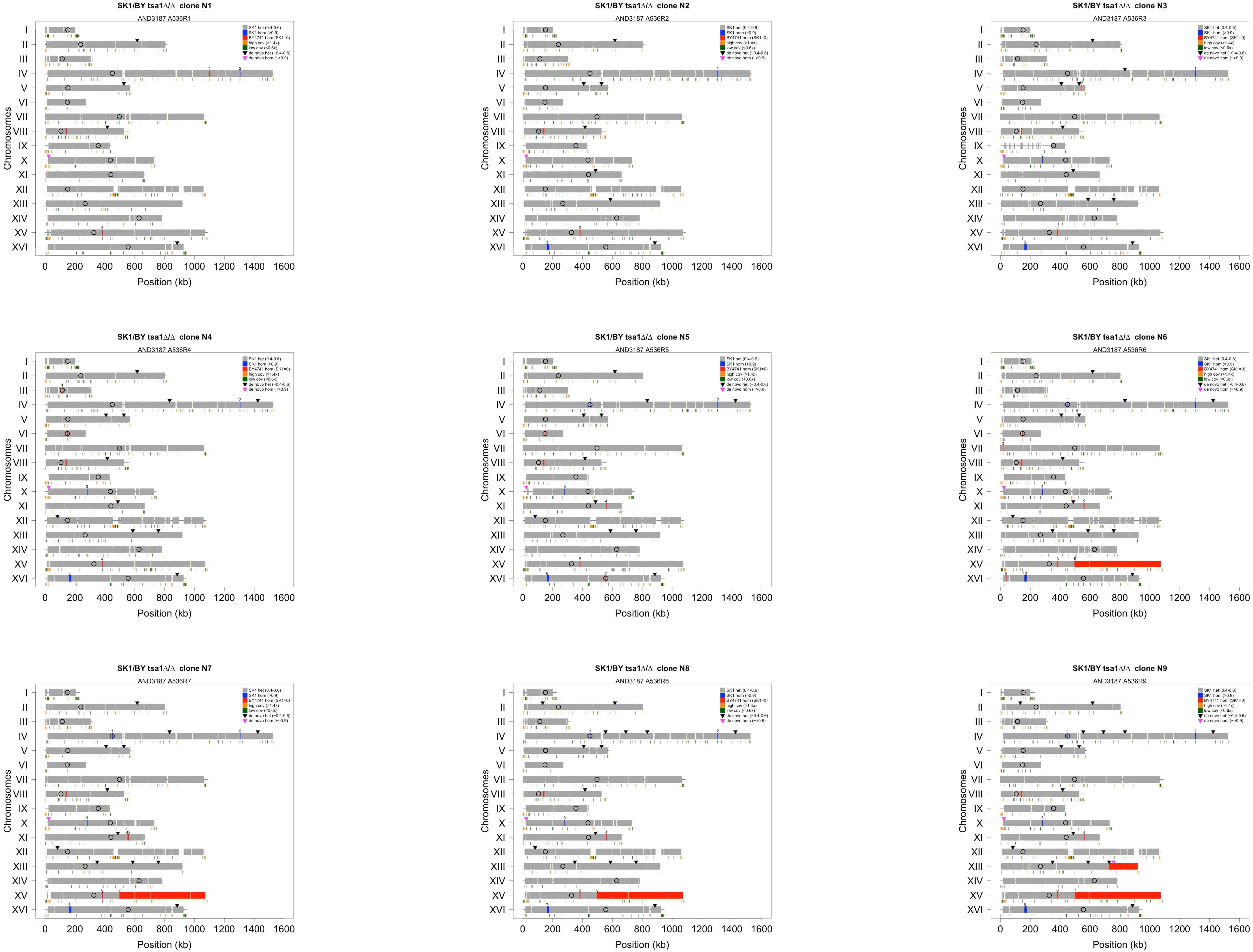
SK1/BY tsa1Δ/Δ clone N1
AND3187 A536R1
SK1/BY tsa1Δ/Δ clone N2
AND3187 A536R2
SK1/BY tsa1Δ/Δ clone N3
AND3187 A536R3
SK1/BY tsa1Δ/Δ clone N4
AND3187 A536R4
SK1/BY tsa1Δ/Δ clone N5
AND3187 A536R5
SK1/BY tsa1Δ/Δ clone N6
AND3187 A536R6
SK1/BY tsa1Δ/Δ clone N7
AND3187 A536R7
SK1/BY tsa1Δ/Δ clone N8
AND3187 A536R8
SK1/BY tsa1Δ/Δ clone N9
AND3187 A536R9
SK1 het (0.4-0.6)
SK1 hom (>0.9)
BY4741 hom (SK1=0)
high cov (>1.4x)
low cov (<0.6x)
de novo het (~0.4-0.6)
de novo hom (~>0.9)
Chromosomes
I
II
III
IV
V
VI
VII
VIII
IX
X
XI
XII
XIII
XIV
XV
XVI
0 200 400 600 800 1000 1200 1400 1600
Position (kb)

Figure S5 (continue)

B

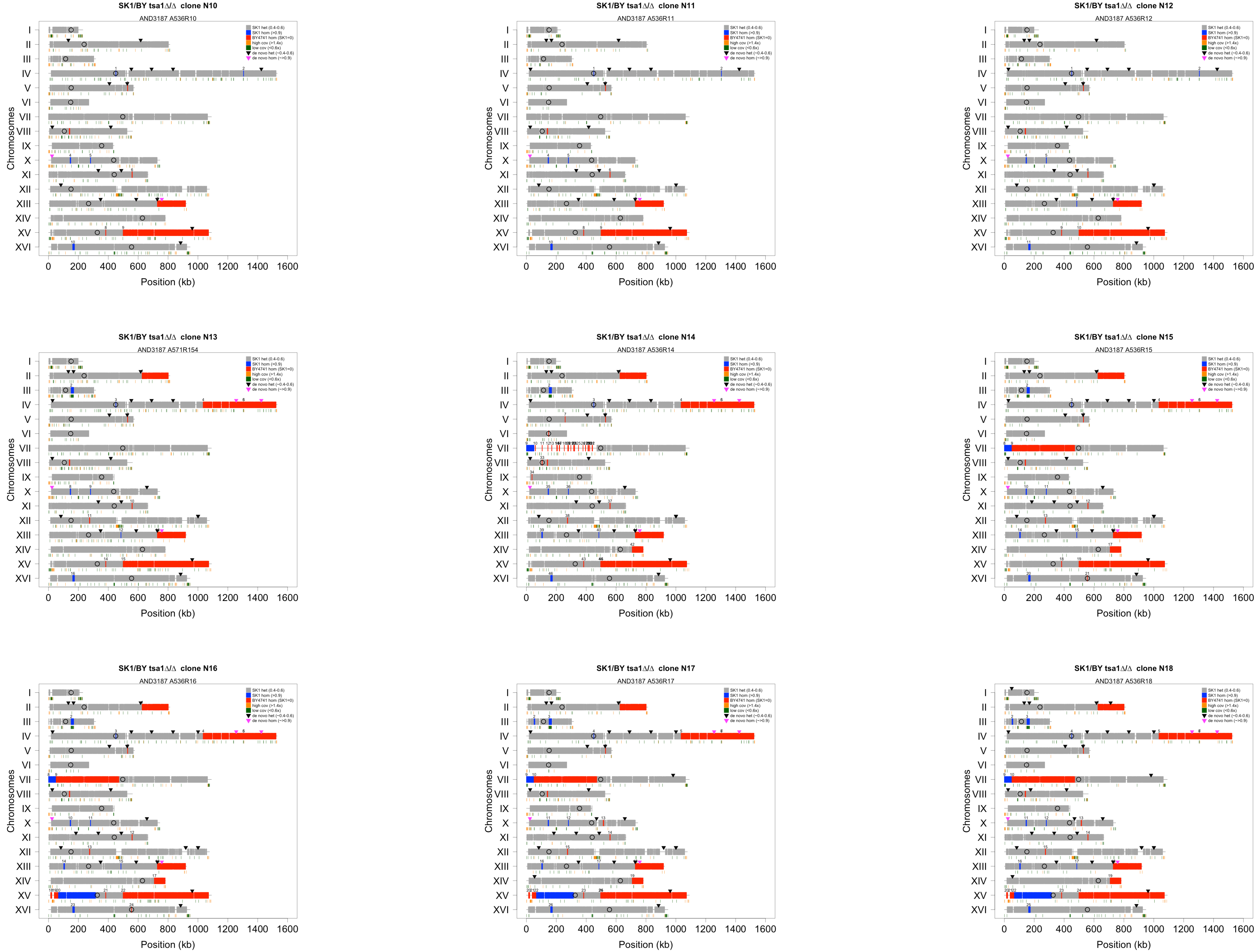

Figure S5 (continue)

B

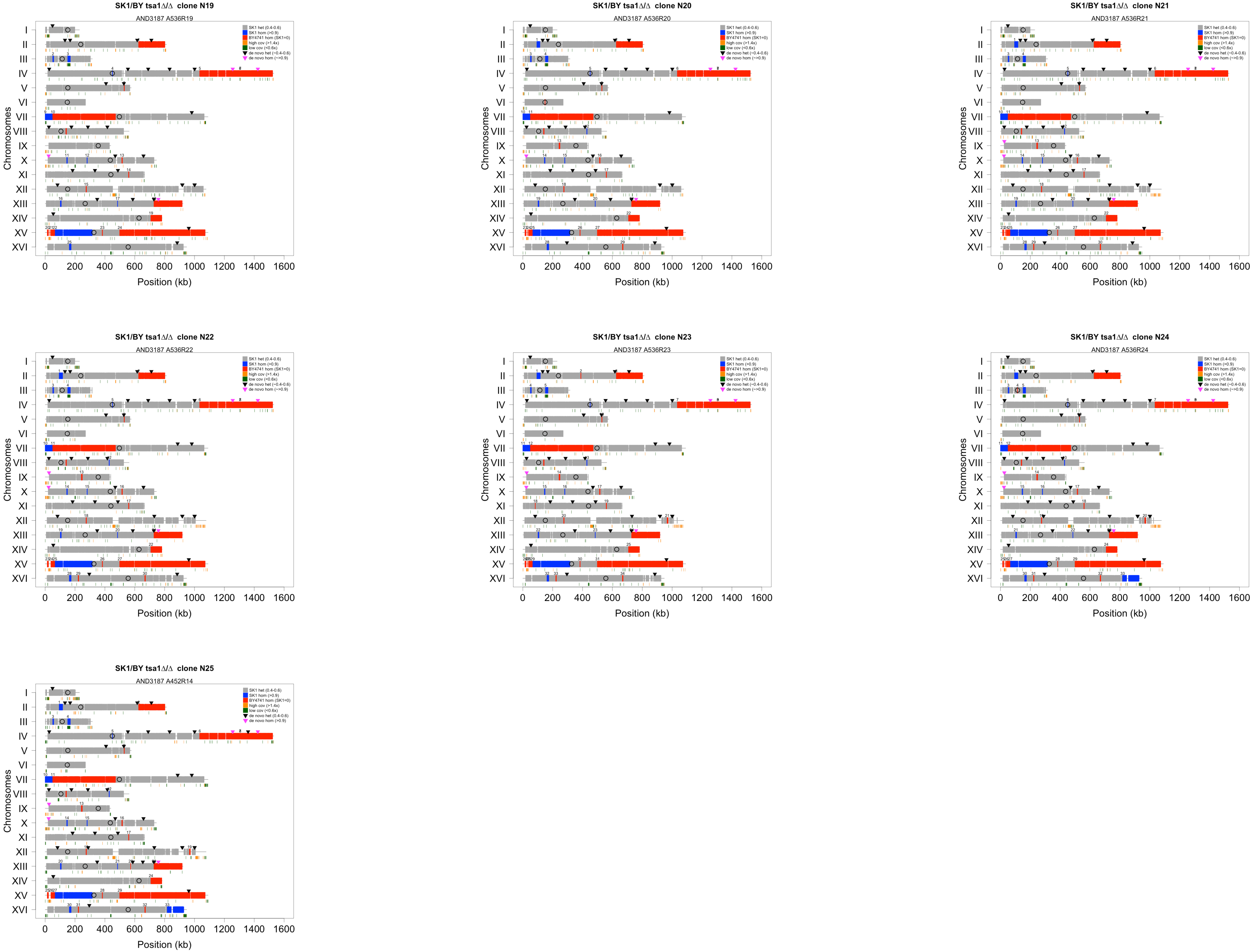

Figure S5

C

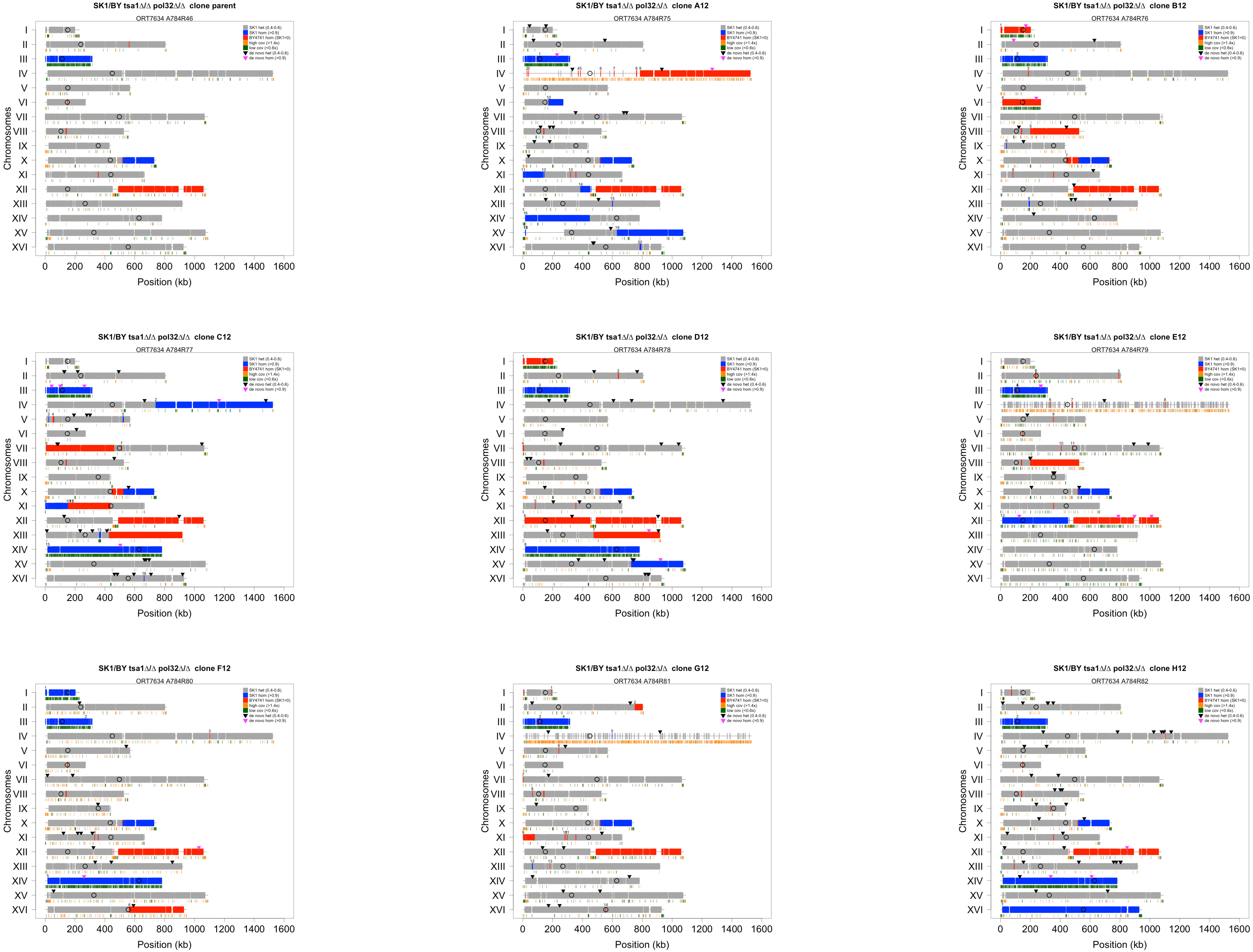
SK1/BY tsa1Δ/Δ pol32Δ/Δ clone parent
ORT7634 A784R46
SK1/BY tsa1Δ/Δ pol32Δ/Δ clone A12
ORT7634 A784R75
SK1/BY tsa1Δ/Δ pol32Δ/Δ clone B12
ORT7634 A784R76
SK1/BY tsa1Δ/Δ pol32Δ/Δ clone C12
ORT7634 A784R77
SK1/BY tsa1Δ/Δ pol32Δ/Δ clone D12
ORT7634 A784R78
SK1/BY tsa1Δ/Δ pol32Δ/Δ clone E12
ORT7634 A784R79
SK1/BY tsa1Δ/Δ pol32Δ/Δ clone F12
ORT7634 A784R80
SK1/BY tsa1Δ/Δ pol32Δ/Δ clone G12
ORT7634 A784R81
SK1/BY tsa1Δ/Δ pol32Δ/Δ clone H12
ORT7634 A784R82
SK1 het (0.4-0.6)
SK1 hom (>0.9)
BY4741 hom (SK1=0)
high cov (>1.4x)
low cov (<0.6x)
de novo het (0.4-0.6)
de novo hom (>0.9)
Chromosomes
I
II
III
IV
V
VI
VII
VIII
IX
X
XI
XII
XIII
XIV
XV
XVI
0
200
400
600
800
1000
1200
1400
1600
Position (kb)

Figure S5 (continue)

C

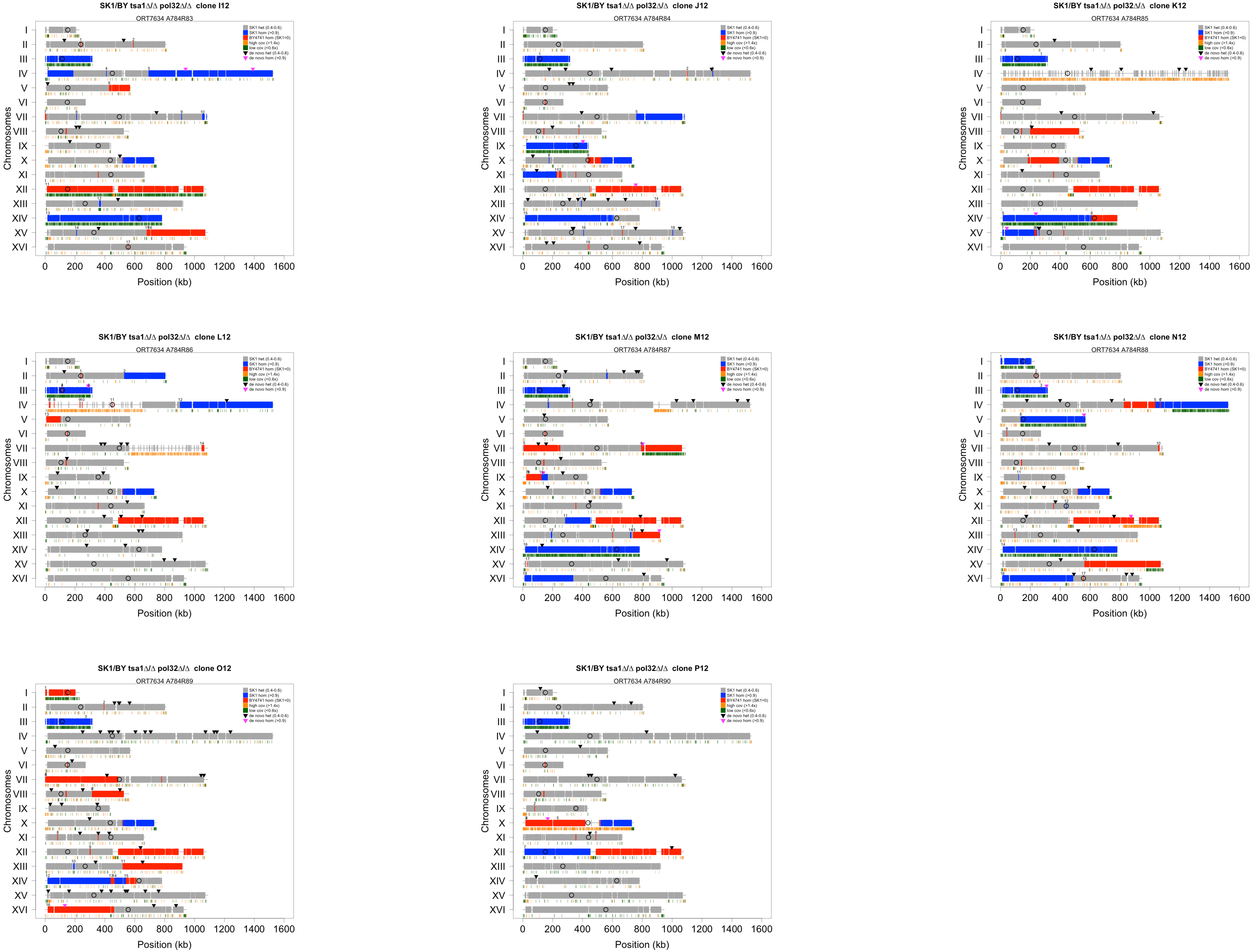

Figure S5

D

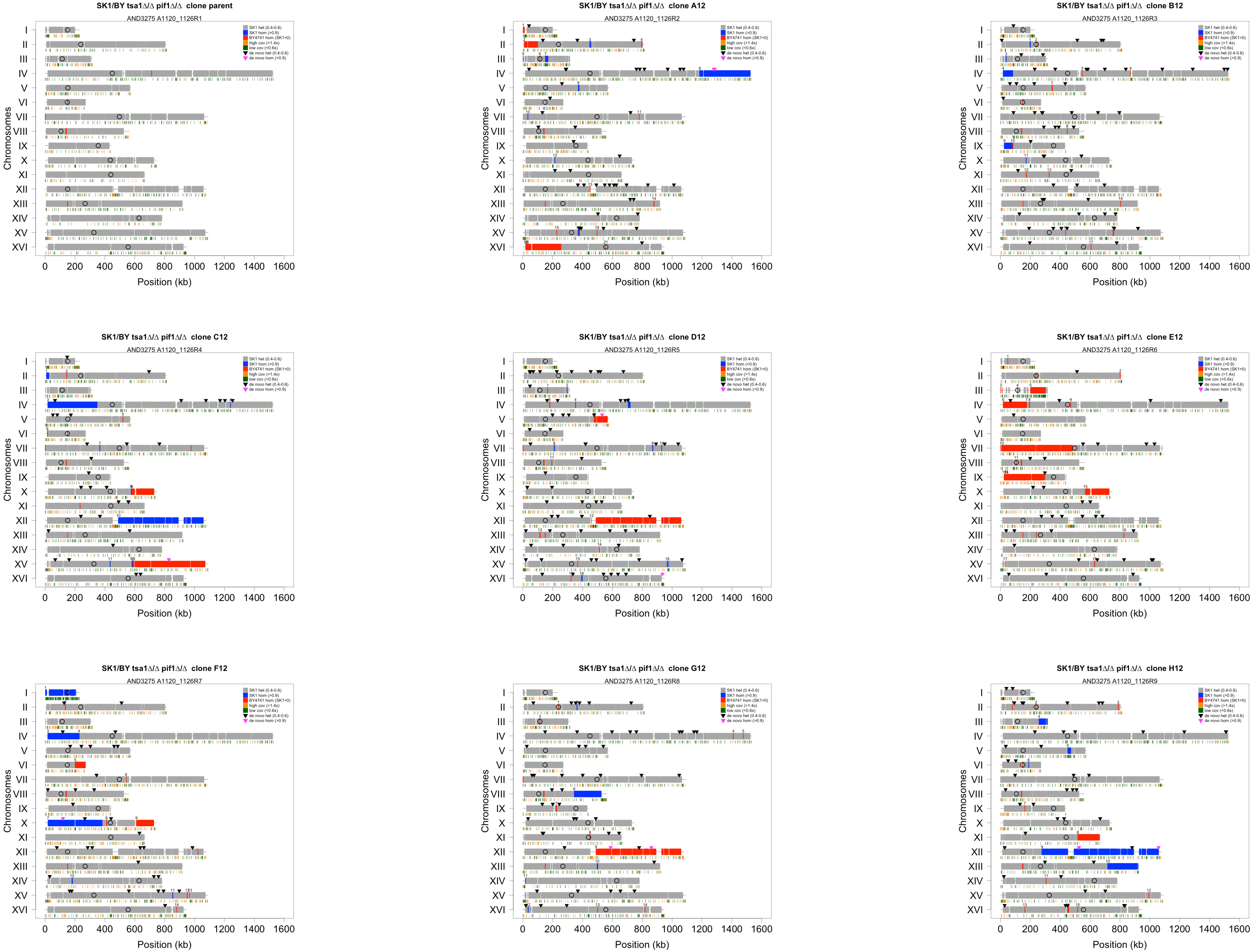

SK1/BY tsa1Δ/Δ pif1Δ/Δ clone parent
AND3275 A1120_1126R1
SK1/BY tsa1Δ/Δ pif1Δ/Δ clone A12
AND3275 A1120_1126R2
SK1/BY tsa1Δ/Δ pif1Δ/Δ clone B12
AND3275 A1120_1126R3
SK1/BY tsa1Δ/Δ pif1Δ/Δ clone C12
AND3275 A1120_1126R4
SK1/BY tsa1Δ/Δ pif1Δ/Δ clone D12
AND3275 A1120_1126R5
SK1/BY tsa1Δ/Δ pif1Δ/Δ clone E12
AND3275 A1120_1126R6
SK1/BY tsa1Δ/Δ pif1Δ/Δ clone F12
AND3275 A1120_1126R7
SK1/BY tsa1Δ/Δ pif1Δ/Δ clone G12
AND3275 A1120_1126R8
SK1/BY tsa1Δ/Δ pif1Δ/Δ clone H12
AND3275 A1120_1126R9
SK1 het (0.4-0.6)
SK1 hom (>0.9)
BY4741 hom (SK1=0)
high cov (>1.4x)
low cov (<0.6x)
de novo het (0.4-0.6)
de novo hom (>0.9)
Chromosomes
I
II
III
IV
V
VI
VII
VIII
IX
X
XI
XII
XIII
XIV
XV
XVI
0 200 400 600 800 1000 1200 1400 1600
Position (kb)

Figure S5 (continue)

D

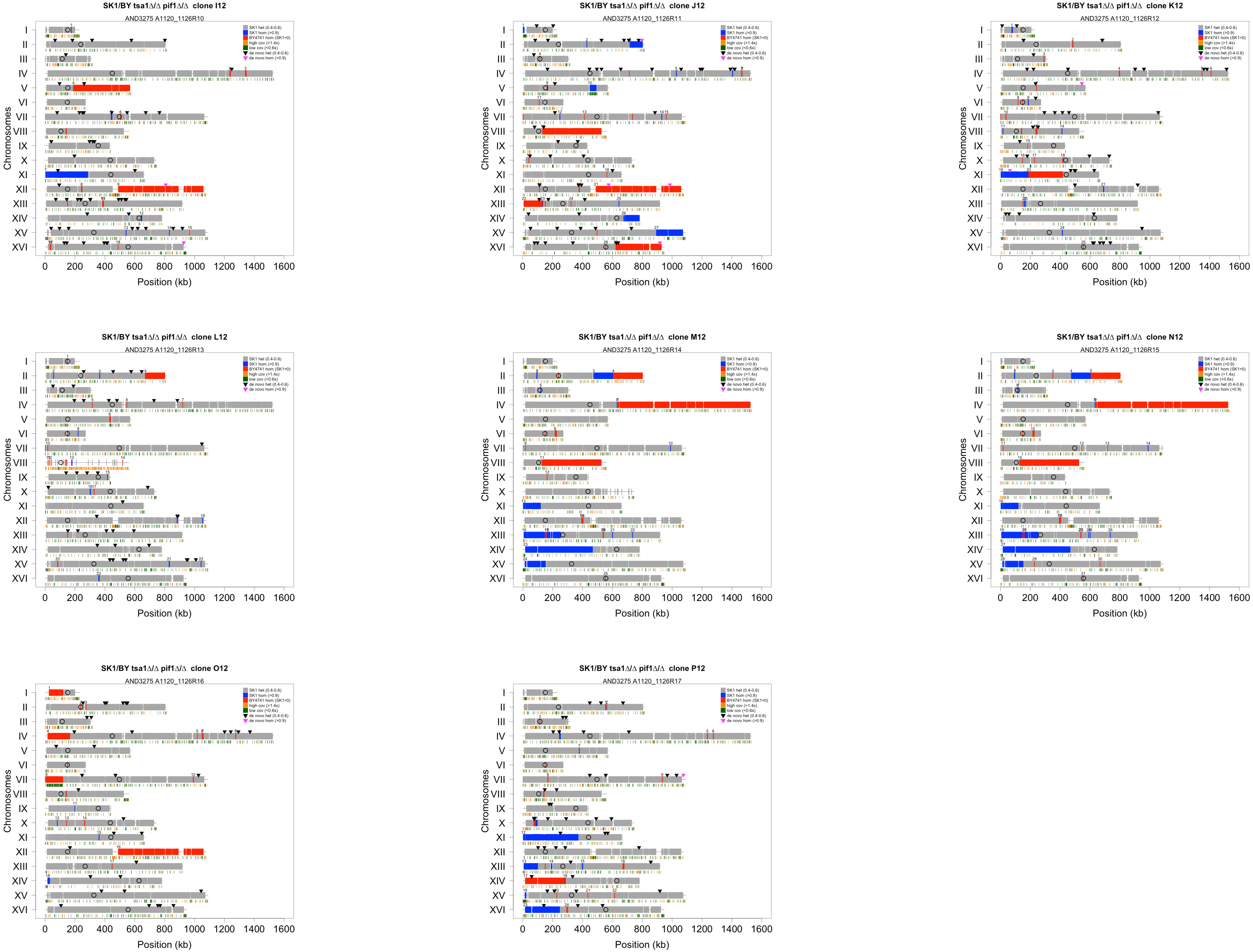

Fig. S5. Genome profile of the SK1/BY *tsa1* MA lines. The genotype of the 53,523 SK1 *vs.* BY polymorphisms are plotted on the 16 *S. cerevisiae* chromosomes with different colours according to their allelic ratio (heterozygous SK1/BY in grey, homozygous SK1 in blue and homozygous BY in red). Only regions with ≥3 adjacent markers of the same genotype are shown (see Materials and Methods). The triangles indicate the location of the heterozygous (black) or "homozygous" (purple) *de novo* mutations (SNP, MNP, complex and small indels, see Materials and Methods). Green and orange bars are 1 kb windows with ratio of mean depth coverage <0.6 or >1.4 compared with mean depth coverage of the sample, respectively. Numbers indicate the LOH region ID as reported in Supplemental Table 12. Strain name, NGS clones name (Datasets S1 and 2) and data are reported for each clone. (*A*) Parental and fifteen SK1/BY *tsa1* clones at passage 25. (*B*) SK1/BY *tsa1* clone N from passage 1 to 25. (*C*) Parental and sixteen SK1/BY *tsa1 pol32* clones at passage 12. (*D*) Parental and fifteen SK1/BY *tsa1 pif1* clones at passage 12.

Figure S6

A

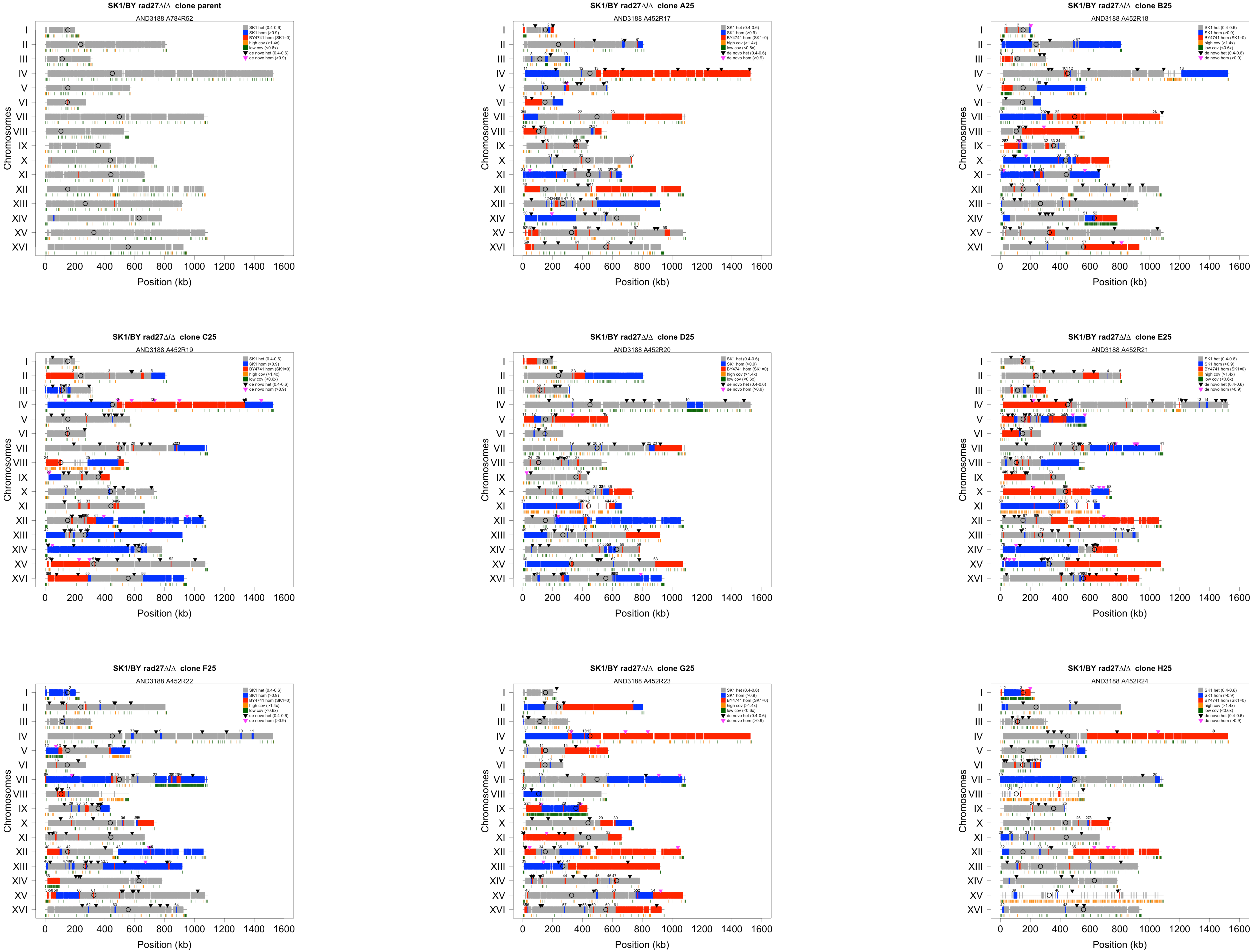
SK1/BY rad27Δ/Δ clone parent
AND3188 A784R52
SK1/BY rad27Δ/Δ clone A25
AND3188 A452R17
SK1/BY rad27Δ/Δ clone B25
AND3188 A452R18
SK1/BY rad27Δ/Δ clone C25
AND3188 A452R19
SK1/BY rad27Δ/Δ clone D25
AND3188 A452R20
SK1/BY rad27Δ/Δ clone E25
AND3188 A452R21
SK1/BY rad27Δ/Δ clone F25
AND3188 A452R22
SK1/BY rad27Δ/Δ clone G25
AND3188 A452R23
SK1/BY rad27Δ/Δ clone H25
AND3188 A452R24
SK1 het (0.4-0.6)
SK1 hom (>0.9)
BY4741 hom (SK1=0)
high cov (>1.4x)
low cov (<0.6x)
de novo het (0.4-0.6)
de novo hom (>0.9)
Chromosomes
I
II
III
IV
V
VI
VII
VIII
IX
X
XI
XII
XIII
XIV
XV
XVI
Position (kb)
0
200
400
600
800
1000
1200
1400
1600

Figure S6 (continue)

A

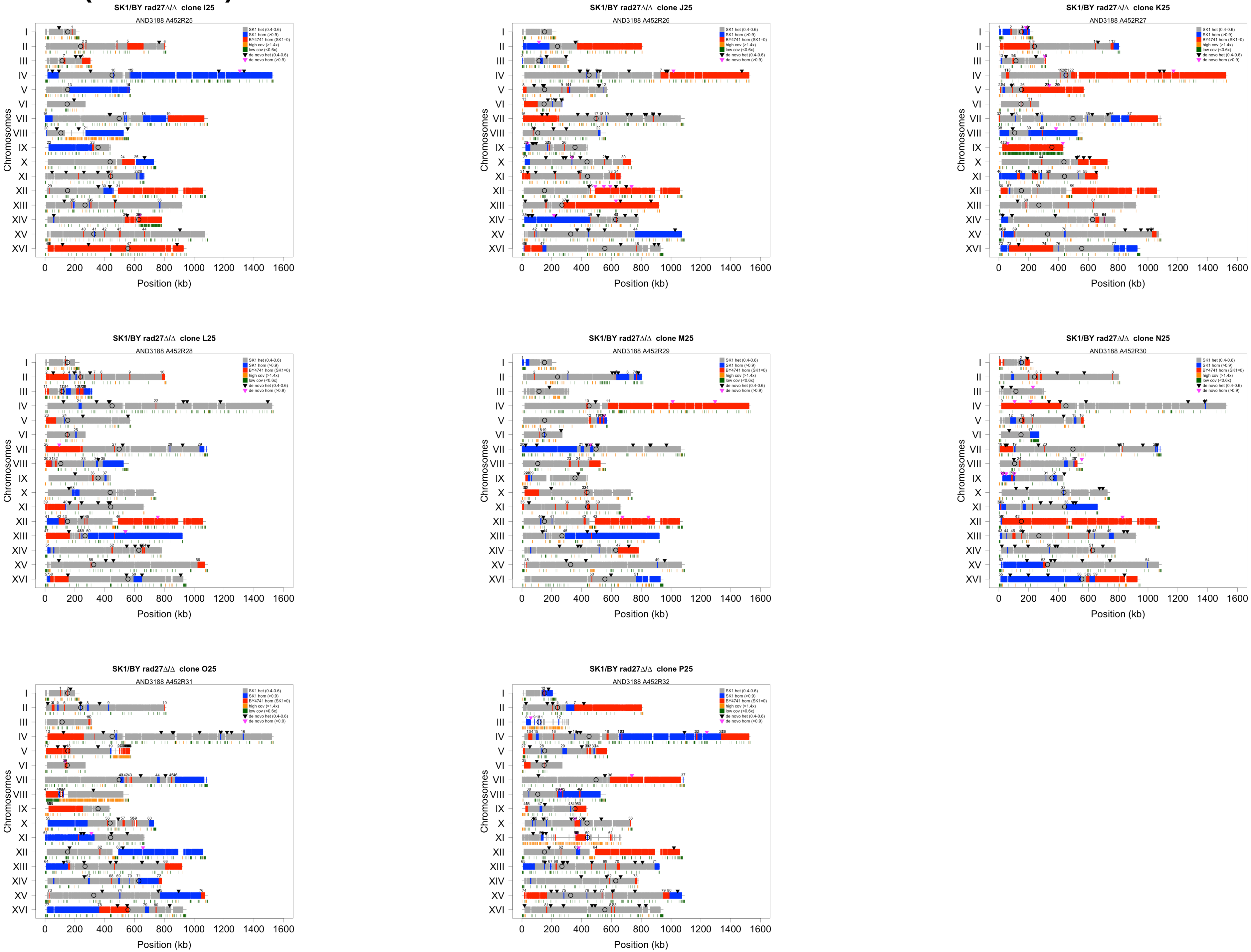

Figure S6

B

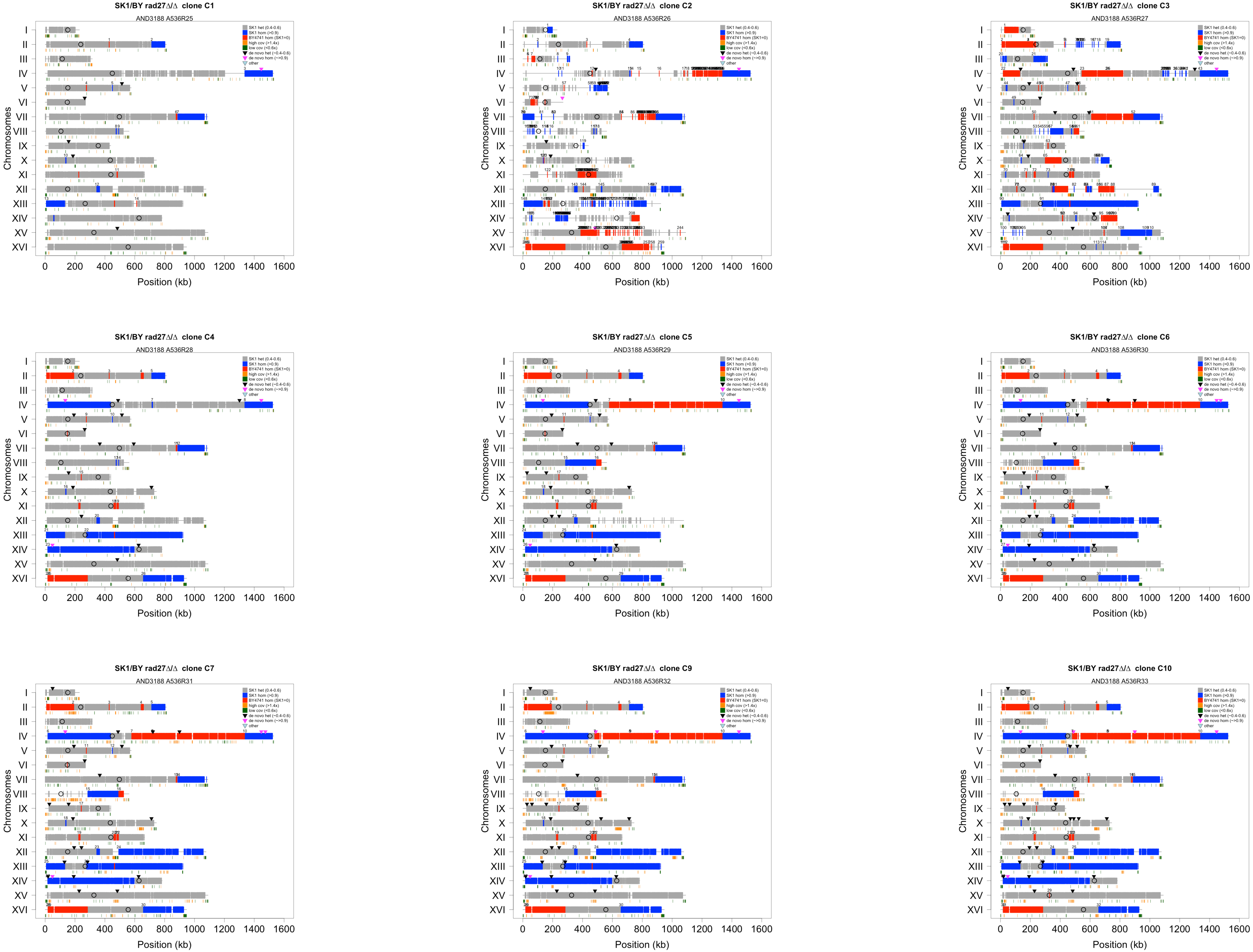
SK1/BY rad27Δ/Δ clone C1
AND3188 A536R25
SK1/BY rad27Δ/Δ clone C2
AND3188 A536R26
SK1/BY rad27Δ/Δ clone C3
AND3188 A536R27
SK1/BY rad27Δ/Δ clone C4
AND3188 A536R28
SK1/BY rad27Δ/Δ clone C5
AND3188 A536R29
SK1/BY rad27Δ/Δ clone C6
AND3188 A536R30
SK1/BY rad27Δ/Δ clone C7
AND3188 A536R31
SK1/BY rad27Δ/Δ clone C9
AND3188 A536R32
SK1/BY rad27Δ/Δ clone C10
AND3188 A536R33
SK1 het (0.4-0.6)
SK1 hom (>0.9)
BY4741 hom (SK1=0)
high cov (>1.4x)
low cov (<0.6x)
de novo het (~0.4-0.6)
de novo hom (~>0.9)
other
Chromosomes
I
II
III
IV
V
VI
VII
VIII
IX
X
XI
XII
XIII
XIV
XV
XVI
0
200
400
600
800
1000
1200
1400
1600
Position (kb)

Figure S6 (continue)

B

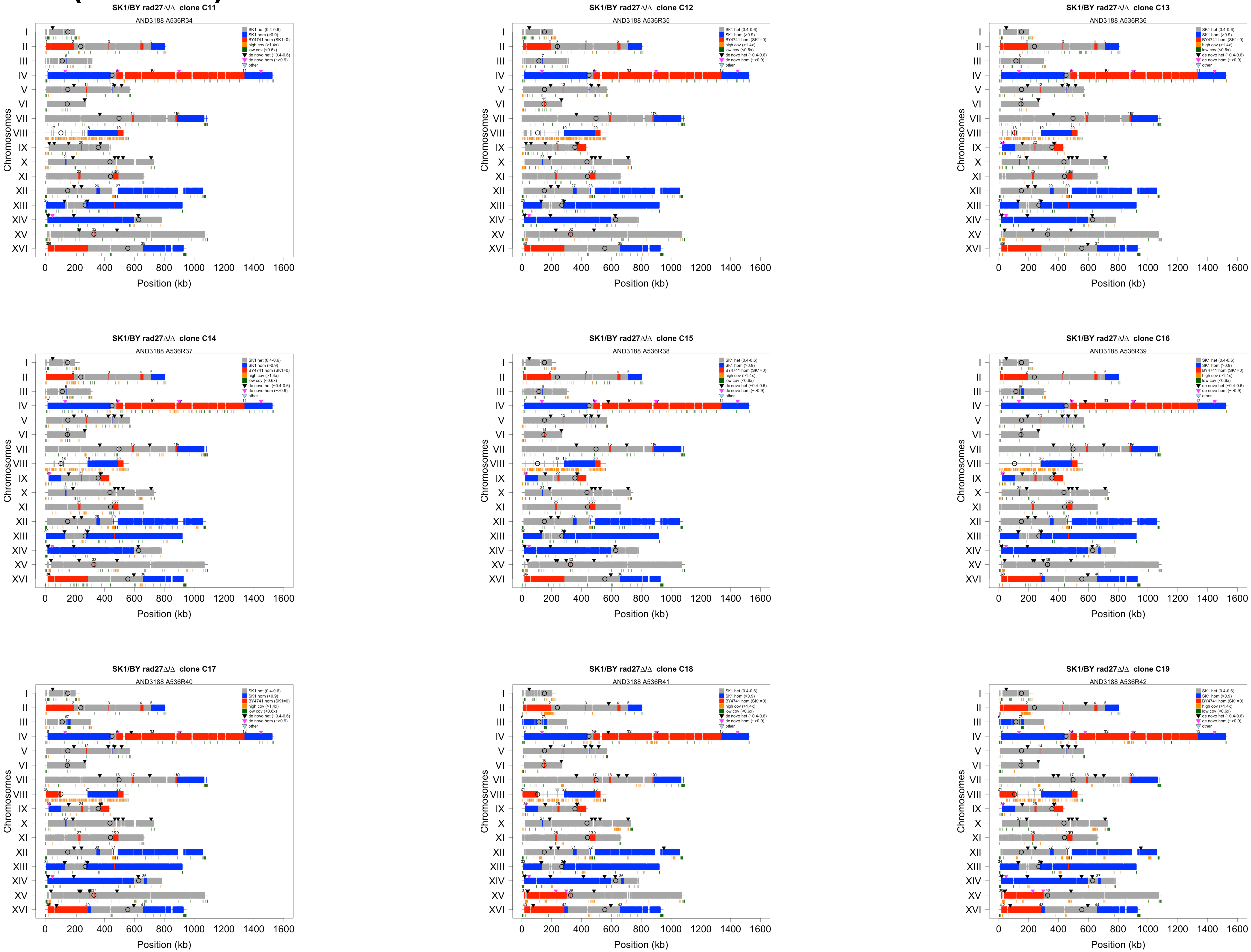

Figure S6 (continue)

B

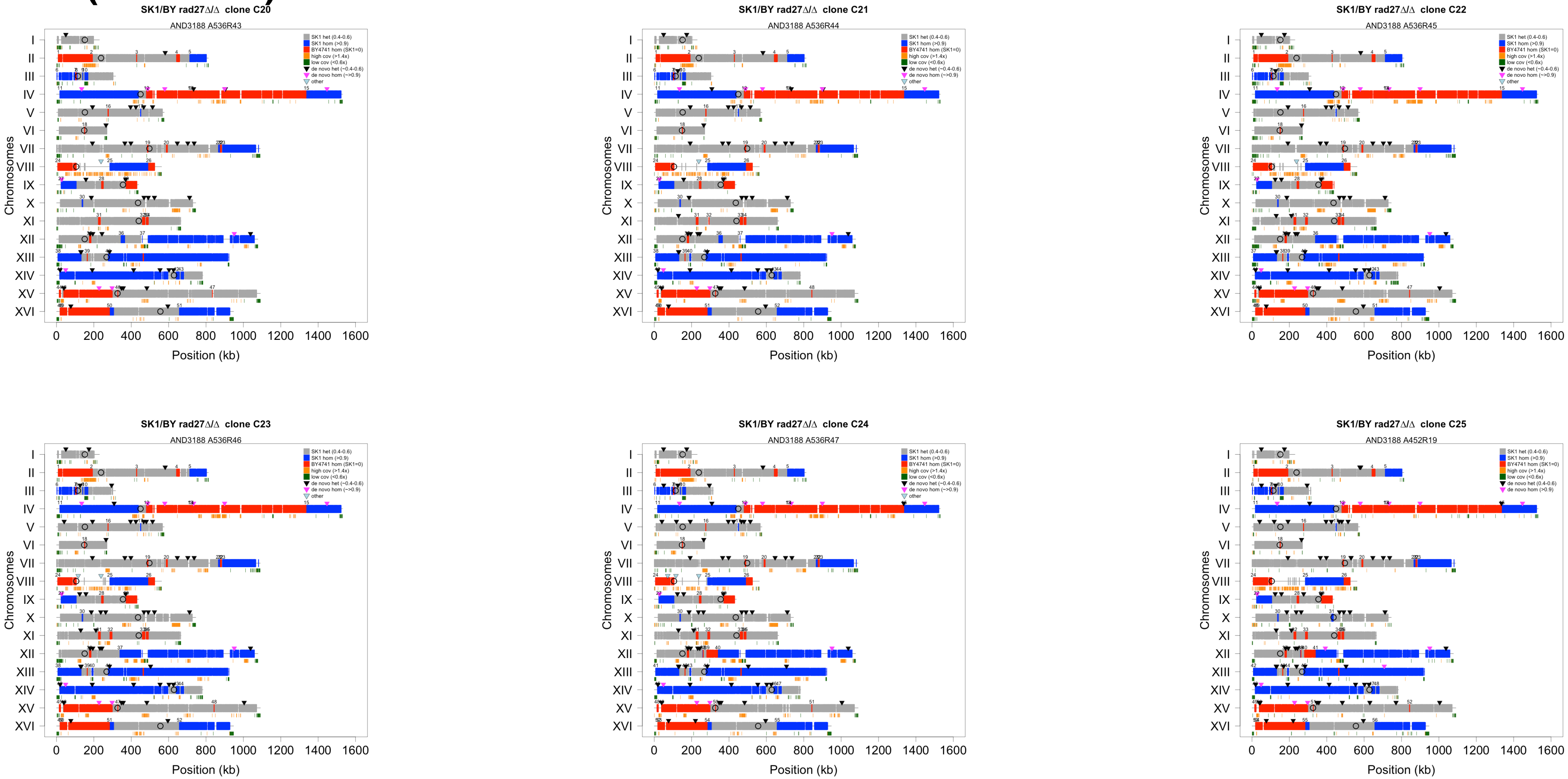

Fig. S6. Genome profile of the SK1/BY *rad27* MA lines. (*A*) Parental and sixteen SK1/BY *rad27* clones at passage 25. (*B*) SK1/BY *rad27* clone C from passage 1 to 25, (see *SI Appendix*, Fig. S5 for legends). Complex and multiple LOH calls were observed on several chromosomes at passages 2 and 3. But, the lineage between passage 1 and 4 remained consistent with the appearance and maintenance of several *de novo* heterozygous SNP and LOH (for examples, chr. IV: 1447,338 G->GA and chr. X: 186,255 T->C mutations and chr. XIII and XVI LOHs) which arose at passage 1. Also, unusual variability of allelic ratio of SK1 markers were observed beyond the threshold 0.4-0.6 and 0.9-1.0 suggesting transient chromosome ploidy variations and likely colony mosaicism that prevented to definitely ascertain the cell genotypes.

Figure S7

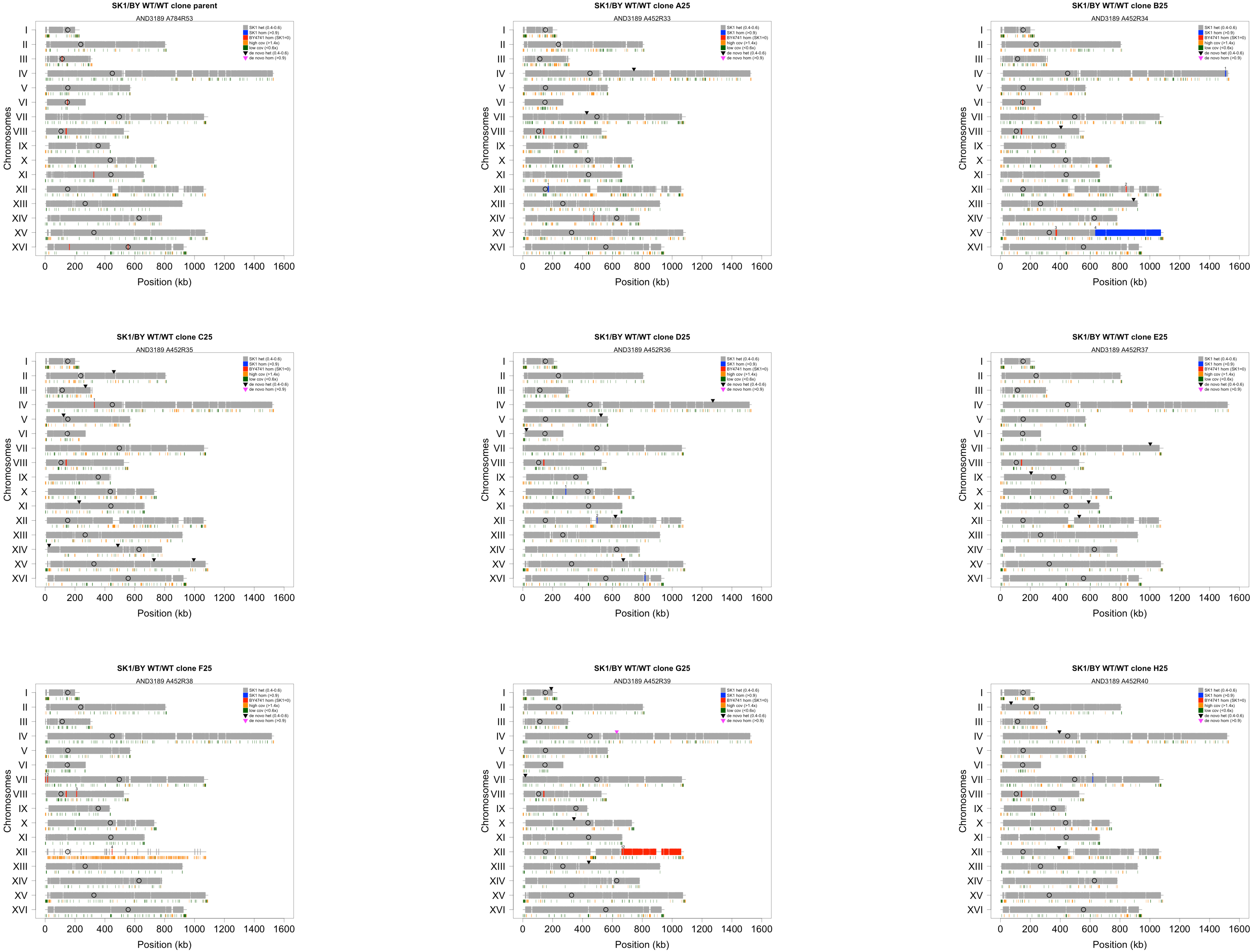
SK1/BY WT/WT clone parent
AND3189 A784R53
SK1/BY WT/WT clone A25
AND3189 A452R33
SK1/BY WT/WT clone B25
AND3189 A452R34
SK1/BY WT/WT clone C25
AND3189 A452R35
SK1/BY WT/WT clone D25
AND3189 A452R36
SK1/BY WT/WT clone E25
AND3189 A452R37
SK1/BY WT/WT clone F25
AND3189 A452R38
SK1/BY WT/WT clone G25
AND3189 A452R39
SK1/BY WT/WT clone H25
AND3189 A452R40
SK1 het (0.4-0.6)
SK1 hom (>0.9)
BY4741 hom (SK1=0)
high cov (>1.4x)
low cov (<0.6x)
de novo het (0.4-0.6)
de novo hom (>0.9)
Chromosomes
I
II
III
IV
V
VI
VII
VIII
IX
X
XI
XII
XIII
XIV
XV
XVI
0
200
400
600
800
1000
1200
1400
1600
Position (kb)

Figure S7 (continue)

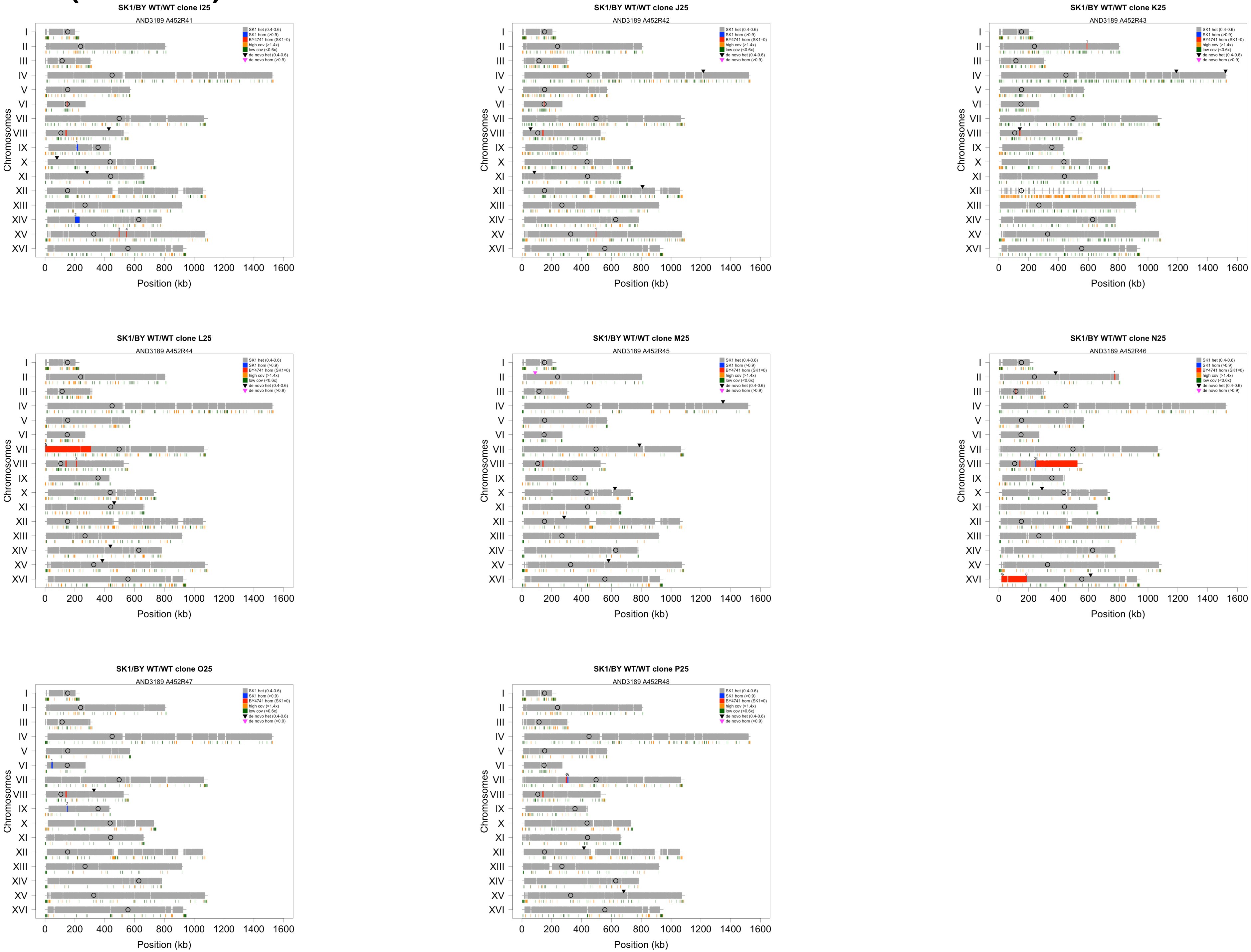

Fig. S7. Genome profiles of parental and sixteen SK1/BY *WT* clones at passage 25. (see *SI Appendix*, Fig. S5 for legends).

Figure S8

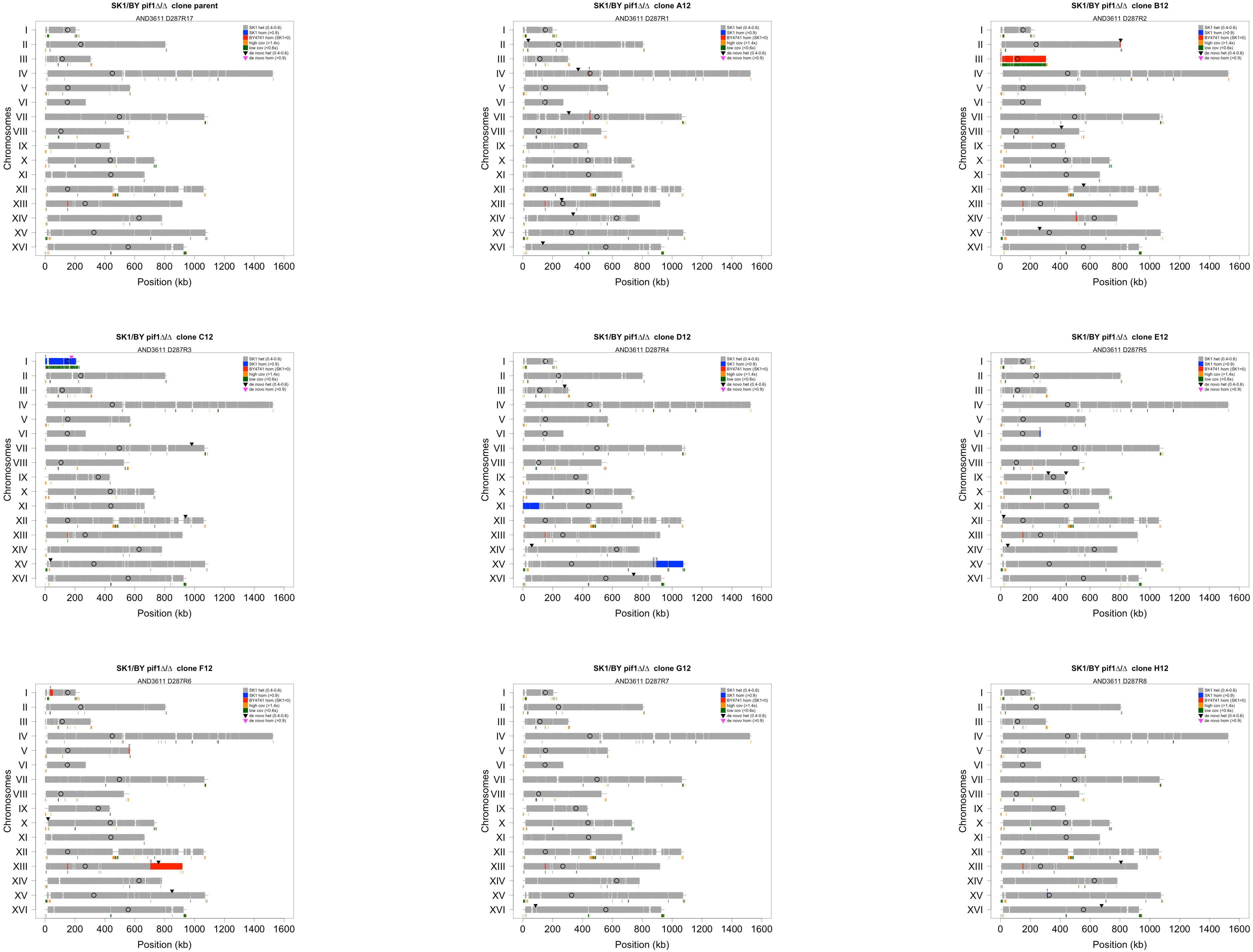
SK1/BY pif1Δ/Δ clone parent
AND3611 D287R17
SK1/BY pif1Δ/Δ clone A12
AND3611 D287R1
SK1/BY pif1Δ/Δ clone B12
AND3611 D287R2
SK1/BY pif1Δ/Δ clone C12
AND3611 D287R3
SK1/BY pif1Δ/Δ clone D12
AND3611 D287R4
SK1/BY pif1Δ/Δ clone E12
AND3611 D287R5
SK1/BY pif1Δ/Δ clone F12
AND3611 D287R6
SK1/BY pif1Δ/Δ clone G12
AND3611 D287R7
SK1/BY pif1Δ/Δ clone H12
AND3611 D287R8
SK1 het (0.4-0.6)
SK1 hom (>0.9)
BY4741 hom (SK1=0)
high cov (>1.4x)
low cov (<0.6x)
de novo het (0.4-0.6)
de novo hom (>0.9)
Chromosomes
I
II
III
IV
V
VI
VII
VIII
IX
X
XI
XII
XIII
XIV
XV
XVI
0
200
400
600
800
1000
1200
1400
1600
Position (kb)

Figure S8 (continue)

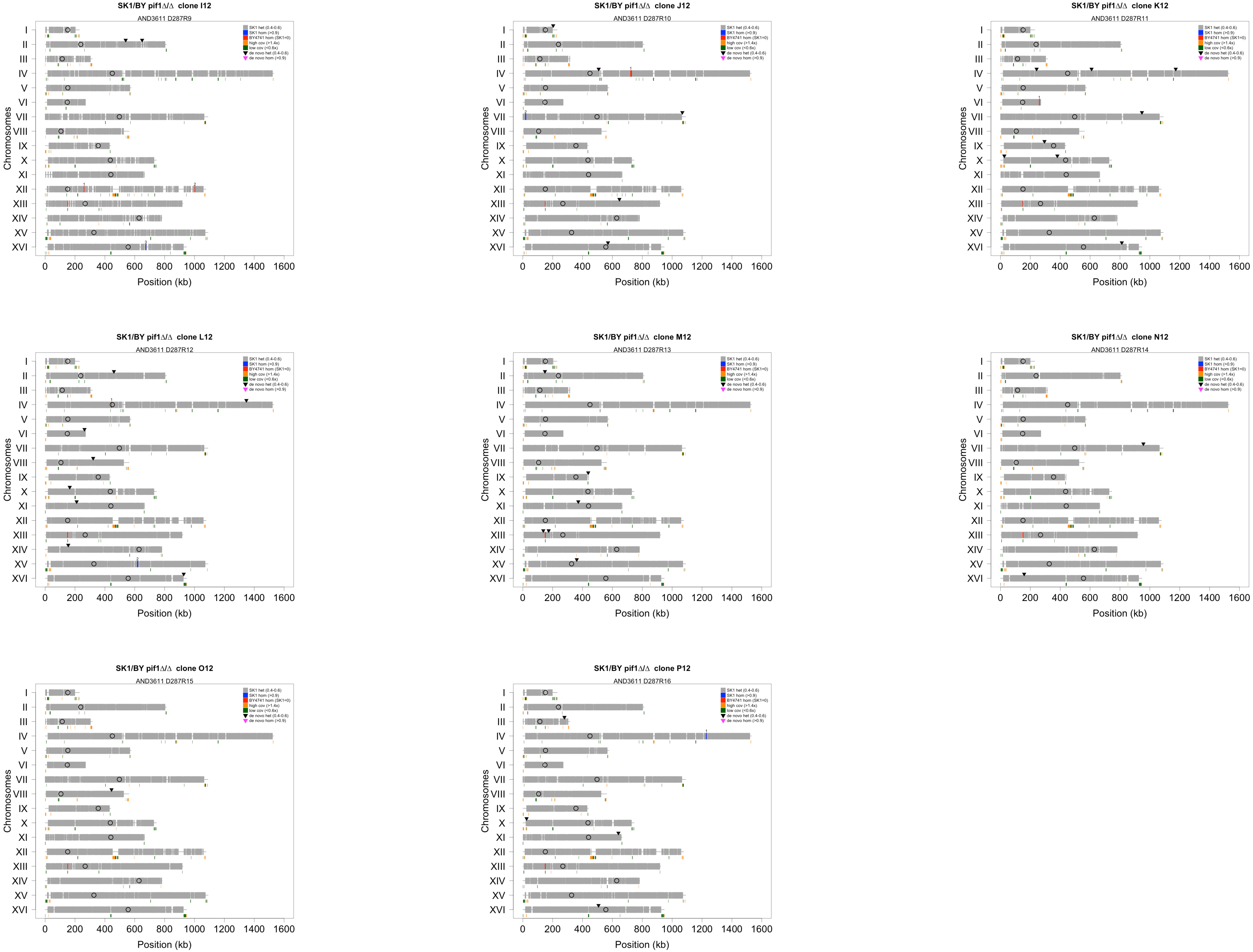

Fig. S8. Genome profiles of parental and sixteen SK1/BY *pif1* clones at passage 12. (see *SI Appendix*, Fig. S5 for legends).

Figure S9

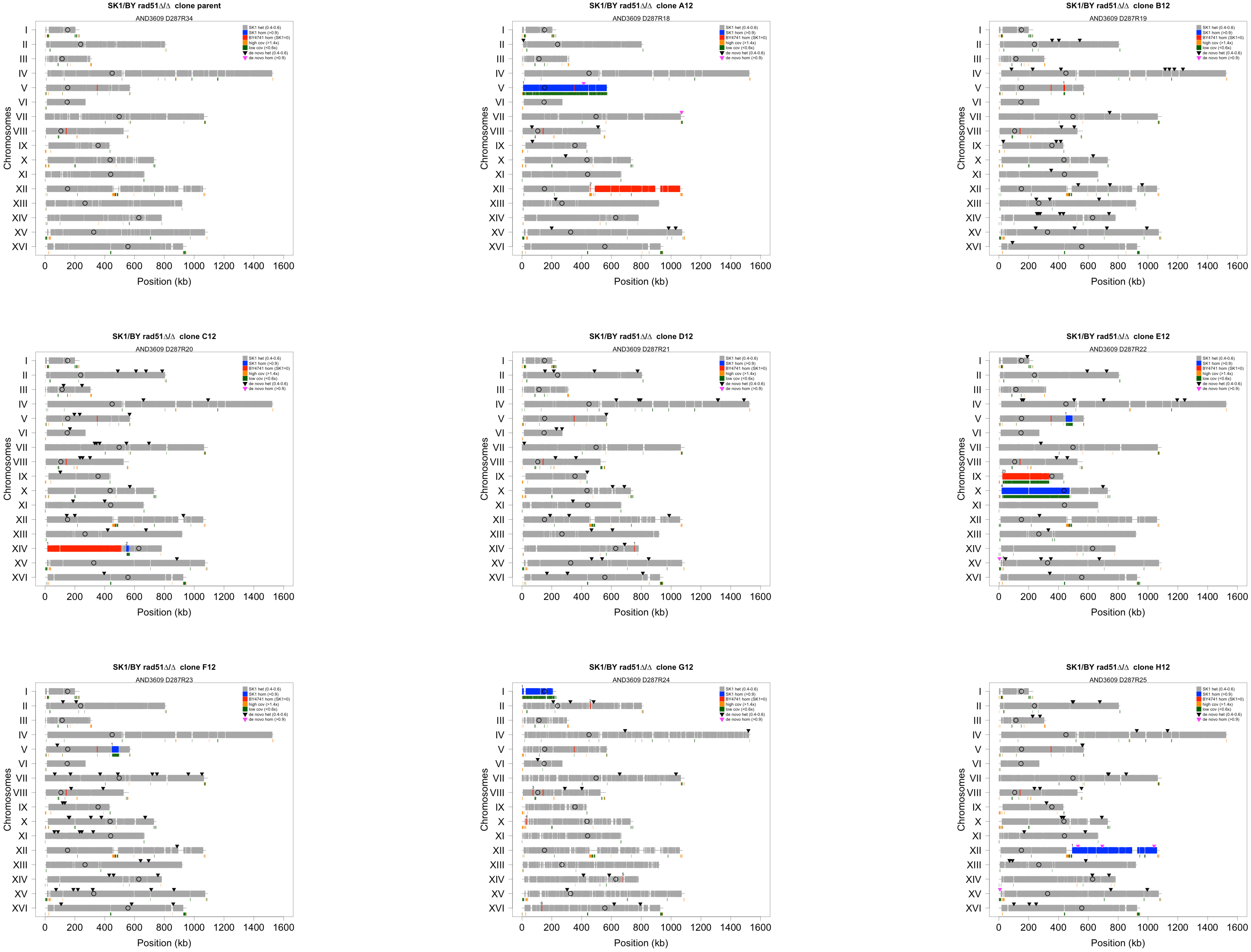
SK1/BY rad51Δ/Δ clone parent
AND3609 D287R34
SK1/BY rad51Δ/Δ clone A12
AND3609 D287R18
SK1/BY rad51Δ/Δ clone B12
AND3609 D287R19
SK1/BY rad51Δ/Δ clone C12
AND3609 D287R20
SK1/BY rad51Δ/Δ clone D12
AND3609 D287R21
SK1/BY rad51Δ/Δ clone E12
AND3609 D287R22
SK1/BY rad51Δ/Δ clone F12
AND3609 D287R23
SK1/BY rad51Δ/Δ clone G12
AND3609 D287R24
SK1/BY rad51Δ/Δ clone H12
AND3609 D287R25
SK1 het (0.4-0.6)
SK1 hom (>0.9)
BY4741 hom (SK1=0)
high cov (>1.4x)
low cov (<0.6x)
de novo het (0.4-0.6)
de novo hom (>0.9)
Chromosomes
I
II
III
IV
V
VI
VII
VIII
IX
X
XI
XII
XIII
XIV
XV
XVI
0
200
400
600
800
1000
1200
1400
1600
Position (kb)

Figure S9 (continue)

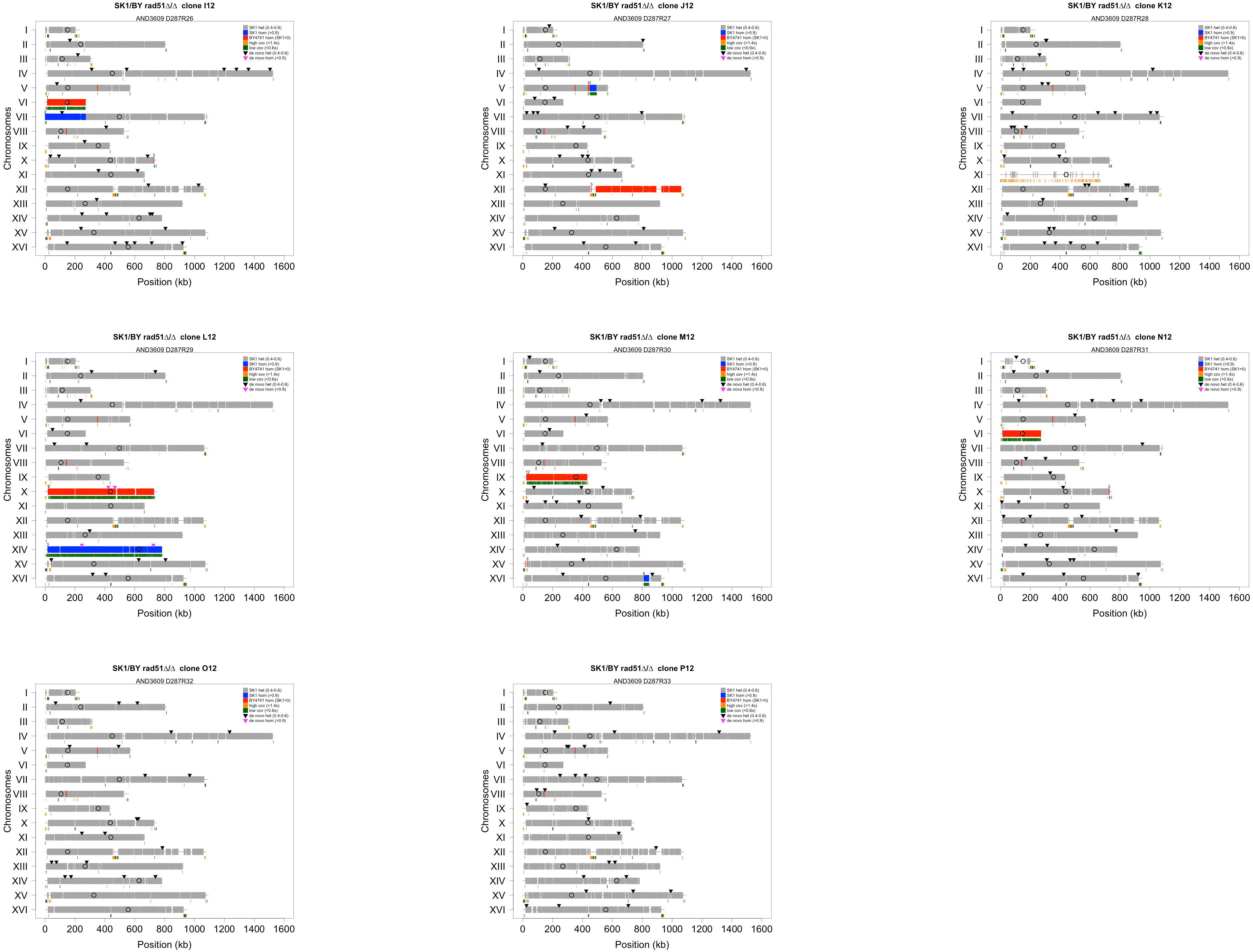

Fig. S9. Genome profiles of parental and sixteen SK1/BY *rad51* clones at passage 12. (see *SI Appendix*, Fig. S5 for legends)

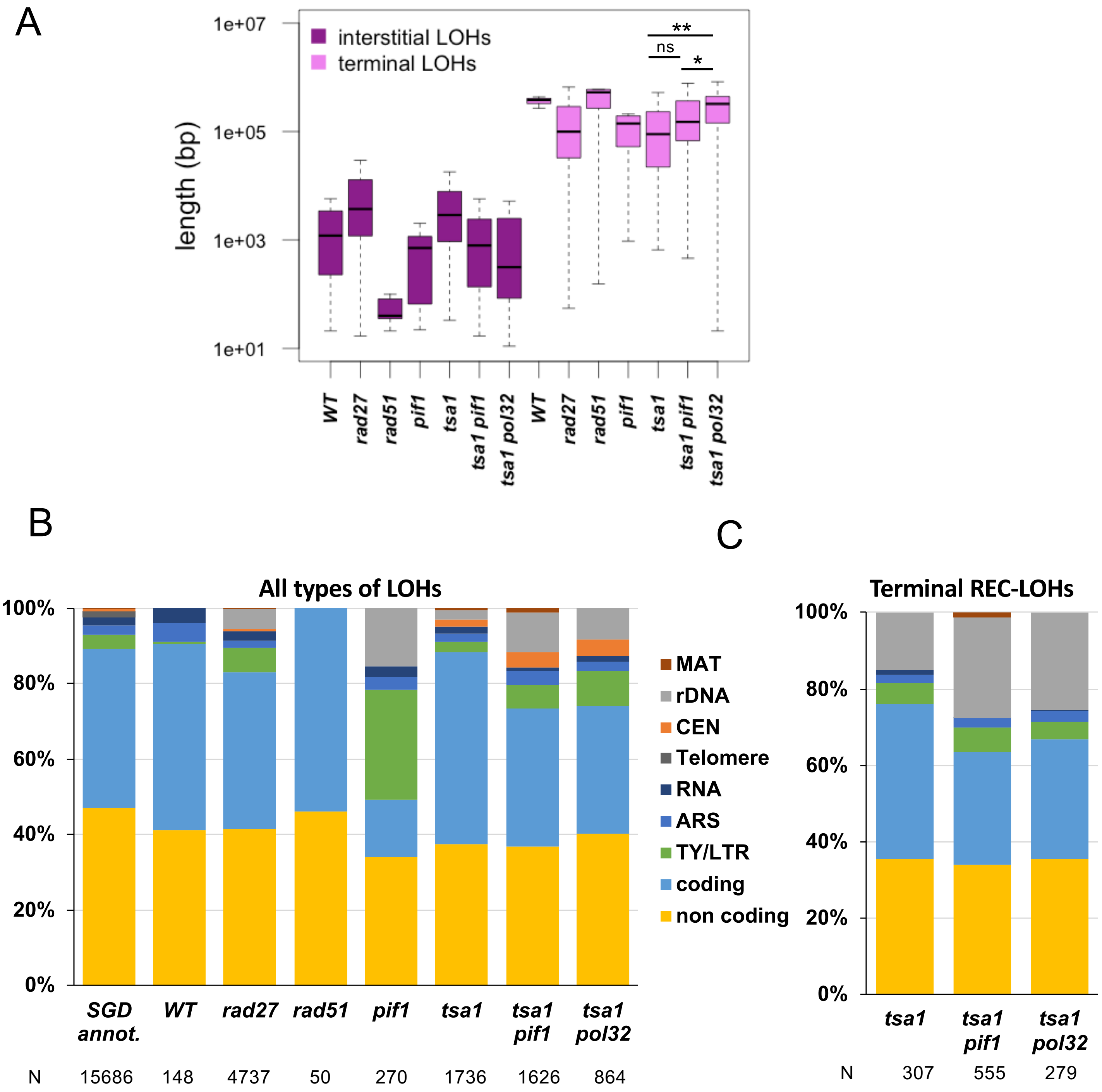

Fig. S10. (*A*) **Size distribution of the interstitial and terminal LOHs.** ns: non significative; *p-value<0.05; **p-value<0.01 (Mann-Whitney-Wilcoxon test). LOHs due to chromosome loss or deletion are not considered. (*B*) **Annotations of LOHs junctions regions.** N: Number of annotations found for each mutant. (*C*) **Annotations of terminal LOHs junctions regions in *tsa1* diploids.** N: Number of annotations found for each mutant. LOHs due to chromosome loss or deletion are not considered.

Movie S1 (separate file). Genome profile evolution in the SK1/BY *tsa1* clone N, from passage 1 to 25. *De novo* mutations and LOH profiles from *SI Appendix*, Fig. S5 are successively shown from the parent clone to the 25th final passage. Legends as in *SI Appendix*, Fig. S5.

Movie S2 (separate file). Genome profile evolution in the SK1/BY *rad27* clone C, from passage 1 to 25. *De novo* mutations and LOH profiles from *SI Appendix*, Fig. S6. Legends as *SI Appendix*, Fig. S6.

Dataset S1 (separate file). Strains used in this study.

Dataset S2 (separate file). Sequencing coverage of individual clones. [a]Name of the clone followed by the number of passages in the MA line. [b]Average of the mean depth coverage values calculated from all chromosomes except the mitochondrial DNA. [c]Threshold of depth of coverage (DP) applied to Freebayes variant calling.

Dataset S3 (separate file). List of small variants identified in the BY/BY diploid strains. [a]Name of the clone followed by the number of passages in the MA line. [b]HET, heterozygous *de novo* mutation; HOM, homozygous *de novo* mutations. [c]SnpEff annotation results. [d]25 bp downstream and upstream sequence surrounding the mutation (small case).

Dataset S4 (separate file). List of small variants identified in the SK1/BY diploid strains. [a]Name of the clone followed by the number of passages in the MA line. [b]HET, heterozygous *de novo* mutation; HOM, homozygous *de novo* mutations. [c]When the *de novo* mutation is located into LOH, the columns show LOH ID, LOH allele and LOH type (see Dataset S12),

NA, if not embedded in a LOH. [d]SK1 polymorphic marker ID (corresponding to chromosome number, position, sequence in the SGD reference genome and sequence in SK1) of the upstream and downstream markers immediately flanking the *de novo* mutation. [e]Allelic ratio of the upstream and downstream markers flanking the *de novo* mutation. [f]SnpEff annotation results. [g]25 bp downstream and upstream sequence surrounding the mutation (small case).

Dataset S5 (separate file). Annotation of mutational hotspots: variants common to more than one clone in BY/BY or SK1/BY diploid mutators. [a]Name of the clone followed by the number of passages in the MA line. [b]25 bp downstream and upstream sequence surrounding the mutation (small case). [c]Indel mutations within a homopolymer or di- or trinucleotide repeated sequence (≥3 repetitions) or within a non-repeated nucleotide motif.

Dataset S6 (separate file). Annotation of mutational hotspots: variant positions with 2 different mutations (ALT1 and ALT2) in the same BY/BY or SK1/BY MA line. Legends as in **c**.

Dataset S7 (separate file). Detection of aneuploidies in the BY/BY or SK1/BY MA lines. [a]Name of the clone followed by the number of passages in the MA line. [b]Average of mean depth coverage at each position, for the indicated chromosome. For chr. XII, the rDNA region (451575-468931) is excluded from the calculation. [c]Loss and gain of chromosomes are indicated in green and red, respectively.

Dataset S8 (separate file). List of structural variants (SVs) in the BY/BY or SK1/BY MA lines. [a]Name of the clone followed by the number of passages in the MA line. [b]Mean depth coverage of the SV region (from start to end position) divided by the sample genome mean

depth coverage. [c]same calculation as in [b] for parent. [d]ratio of [b] and [c] (see Materials and Methods).

Dataset S9 (separate file). Number of mutations per clone. [a]Heterozygous or homozygous SNP, small indels, MNP and complex mutations (detailed in Datasets S3 and 4) specific of one clone, [b]Mutations occurring in more than one clone (detailed in Dataset S5), [c]Number of positions with 2 calls (detailed in Dataset S6), [d]Number of lost or gained chromosomes (detailed in Dataset S7) and [e]Number of Structural Variants (detailed in Dataset S8).

Dataset S10 (separate file). Counts of *de novo* mutations identified in the BY and SK1/BY MA lines. [a]Sum of 2 series of bottlenecks (16 clones at passage 25 for AND2210 and 16 clones at passage 12 for AND3146, strain genotypes in Table S1). AND3146 MA was considered equivalent to 8 clones at passage 25. [b]Number of chromosomes. [c]Number of LOH not associated to a loss of local coverage or whole chromosome loss. [d]Number of LOH associated to a local loss of coverage. [e]Number of LOH associated with chromosome loss. [f]Total number of mutations normalized per clone and passages, then normalized to the WT. [g]Sum of all *de novo* mutational events (mutations detected in a single clone or in >1 clone, positions with 2 calls, lost or gained chromosomes and structural variants).

Dataset S11 (separate file). List of 53,523 SK1 markers used for genome-wide genotyping.

Dataset S12 (separate file). List of LOH in the SK1/BY MA lines. [a]Name of the clone followed by the number of passages in the MA line. [b]Mean SNP markers allelic ratio of LOH region. [c]Copy number of the LOH (see Materials and Methods). [d]REC: LOH not associated to

loss of coverage; DEL: LOH associated with local loss of coverage; chr_loss: LOH associated with chromosome loss. [e]intLOH: interstitial LOH; terLOH: terminal LOH.

Dataset S13 (separate file). List of *de novo* small variants identified in the SK1/BY *tsa1* clone N lineage, from passage 1 to 25. Legends as in Dataset S4.

Dataset S14 (separate file). List of *de novo* small variants in the SK1/BY *rad27* clone C lineage, from passage 1 to 25. Legends as in Dataset S4.

Dataset S15 (separate file). List of LOH in the SK1/BY *tsa1* clone N lineage, from passage 1 to 25. Legends as in Dataset S12.

Dataset S16 (separate file). List of LOH in the SK1/BY *rad27* clone C lineage, from passage 1 to 25. Legends as in Dataset S12.

SI References

1. C. B. Brachmann, *et al.*, Designer deletion strains derived from Saccharomyces cerevisiae S288C: a useful set of strains and plasmids for PCR-mediated gene disruption and other applications. *Yeast* **14**, 115–32 (1998).
2. A. Serero, C. Jubin, S. Loeillet, P. Legoix-Né, A. G. Nicolas, Mutational landscape of yeast mutator strains. *Proc. Natl. Acad. Sci.* **111**, 1897–1902 (2014).
3. E. a Winzeler, *et al.*, Functional characterization of the S. cerevisiae genome by gene deletion and parallel analysis. *Science (80-.).* **285**, 901–6 (1999).
4. R. Laureau, *et al.*, Extensive Recombination of a Yeast Diploid Hybrid through Meiotic Reversion. *PLoS Genet.* **12** (2016).

5. V. Sommermeyer, C. Béneut, E. Chaplais, M. E. Serrentino, V. Borde, Spp1, a member of the Set1 Complex, promotes meiotic DSB formation in promoters by tethering histone H3K4 methylation sites to chromosome axes. *Mol. Cell* **49**, 43–54 (2013).
6. H. Debrauwère, S. Loeillet, W. Lin, J. Lopes, A. Nicolas, Links between replication and recombination in Saccharomyces cerevisiae: A hypersensitive requirement for homologous recombination in the absence of Rad27 activity. *Proc. Natl. Acad. Sci. U. S. A.* **98** (2001).
7. H. Li, R. Durbin, Fast and accurate short read alignment with Burrows-Wheeler transform. *Bioinformatics* **25**, 1754–1760 (2009).
8. D. Meyer, *et al.*, Cooperation between non-essential DNA polymerases contributes to genome stability in Saccharomyces cerevisiae. *DNA Repair (Amst).* **76** (2019).
9. E. Afgan, *et al.*, The Galaxy platform for accessible, reproducible and collaborative biomedical analyses: 2018 update. *Nucleic Acids Res.* **46**, W537–W544 (2018).
10. E. Garrison, G. Marth, Haplotype-based variant detection from short-read sequencing. 1–9 (2012).
11. C. Jubin, A. Serero, S. Loeillet, E. Barillot, A. Nicolas, Sequence Profiling of the Saccharomyces cerevisiae Genome Permits Deconvolution of Unique and Multialigned Reads for Variant Detection. *Genes|Genomes|Genetics* **4**, 707–715 (2014).
12. D. M. R. P. Cingolani, A. Platts, L. L. Wang, M. Coon, T. Nguyen, L. Wang, S. J. Land, X. Lu, A program for annotating and predicting the effects of single nucleotide polymorphisms, SnpEff: SNPs in the genome of Drosophila melanogaster strain w1118 ; iso-2; iso-3. *Fly (Austin).* **6**, 80–92 (2012).
13. M. A. Depristo, *et al.*, A framework for variation discovery and genotyping using next-generation DNA sequencing data. *Nat. Genet.* **43**, 491–501 (2011).
14. V. Boeva, *et al.*, Control-FREEC: A tool for assessing copy number and allelic content

using next-generation sequencing data. *Bioinformatics* **28**, 423–425 (2012).

15. R. M. Layer, C. Chiang, A. R. Quinlan, I. M. Hall, LUMPY: A probabilistic framework for structural variant discovery. *Genome Biol.* **15**, 1–19 (2014).

16. T. Rausch, *et al.*, DELLY : structural variant discovery by integrated paired-end and split-read analysis. *Bioinformatics* **28**, 333–339 (2012).

17. D. C. Jeffares, *et al.*, Transient structural variations have strong effects on quantitative traits and reproductive isolation in fission yeast Daniel. *Nat. Commun.*, 1–11 (2017).

18. F. Blokzijl, R. Janssen, R. van Boxtel, E. Cuppen, MutationalPatterns: Comprehensive genome-wide analysis of mutational processes. *Genome Med.* **10**, 1–11 (2018).

19. F. Puddu, *et al.*, Genome architecture and stability in the Saccharomyces cerevisiae knockout collection. *Nature* (2019).

20. S. A. Lujan, *et al.*, Heterogeneous polymerase fidelity and mismatch repair bias genome variation and composition. *Genome Res.* **24**, 1751–1764 (2014).
